# Molecular Switches—Tools for Imparting Control in Drug Delivery Systems

**DOI:** 10.3389/fchem.2022.859450

**Published:** 2022-03-31

**Authors:** Owen Fitzmaurice, Michał Bartkowski, Silvia Giordani

**Affiliations:** School of Chemical Sciences, Dublin City University (DCU), Dublin, Ireland

**Keywords:** molecular switches, responsive systems, endogenous and exogenous stimuli, drug delivery, photo-switches, pH-switches, optical control, pH triggered release

## Abstract

Cancer is a globally prevalent cause of premature mortality. Of growing interest is the development of novel anticancer therapies and the optimisation of associated risks. Major issues presently facing conventional anticancer therapies include systemic toxicity, poor solubility, membrane permeability, and multidrug resistance Nanocarriers have been employed to address these issues. Nanocarriers encapsulate anticancer drugs, enabling them to bypass biological barriers and minimise their adverse side effects. These drug delivery systems offer extensive benefits as they can be modified to gravitate towards specific environmental conditions. To further enhance the safety and efficacy of these drug carriers, modern developments have included incorporating a molecular switching mechanism into their structure. These molecular switches are responsive to endogenous and exogenous stimuli and may undergo reversible and repeatable conformational changes when activated. The incorporation of molecular switches can, therefore, impart stimuli-responsive drug-release control on a DDS. These stimuli can then be manipulated to offer precise dosage control over the drug release at a specific target site. This review discusses recent developments in the design of DDSs incorporating light and pH-responsive molecular switches as drug release controllers.

## Introduction

Cancer has become the most common cause of premature mortality in America, Europe and Asia, preceded only by cardiovascular diseases in certain countries (Wild et al., 2020). The significant global rise in cancer diagnoses also comes with the emergence of various chemotherapeutic medicines. These agents attack rapidly multiplying cells, but as they are cytotoxic, they can have undesired and adverse side effects. With conventional drug delivery, these toxic chemotherapeutics are typically distributed throughout the entire body to reach their target site. Consequently, these drugs must be administered in doses large enough to reach their therapeutic index, increasing the risk of adverse side effects. In many cases, conventional anticancer drug formulations result in normal cells receiving greater exposure to anticancer drugs than tumorous cells (Patel et al., 2013).

A further issue associated with conventional formulations of anticancer therapeutics is their solubility and membrane permeability (Peer et al., 2007; Liu et al., 2011). Accompanying the increased knowledge of drug-receptor targets, and the advances in fragment-based drug design to match them, is the understandable escalation in the structural complexity of new active pharmaceutical ingredient (API) leads. A notably challenging aspect of drug design is finding the balance between the aqueous solubility of the drug and its membrane permeability (Singh et al., 2011). These parameters are fundamental to the drug’s absorption rate; hence, the extent of its bioavailability. With increasing API size and structural complexity, the difficulty of API solubilisation escalates (Savjani et al., 2012). The problem is readily apparent when ∼70% of new drug candidates show low aqueous solubility; under 100 μg/ml (Ku and Dulin, 2010; Kawabata et al., 2011). Low solubility is a significant issue for anticancer therapeutics, which are often structurally complex molecules. Consequently, the clinical application of these anticancer drugs, which have otherwise excellent therapeutic effects, becomes limited (Liu et al., 2011).

Yet another issue facing conventional anticancer therapies is the emergence of multidrug resistance (MDR). MDR is a defence mechanism against antineoplastic drugs that certain cancer cells may possess. These MDR cancer cells have elevated levels of enzymes, which degrade the therapeutics; as well as membrane-bound proteins, known as MDR-transporters, which efflux anticancer drugs out of the cell (Szakács et al., 2006). A well known MDR-transporter is the P-glycoprotein, which prevents cellular uptake of most anticancer therapeutics (Seelig, 2020). Furthermore, only specific cancer cells may express MDR proteins; as such, conventional chemotherapy often leads to anticancer therapeutics killing non-MDR cells, whilst leaving behind MDR cells. For this reason, chemotherapy often fails with tumour recurrence (Peer et al., 2007).

Fortunately, ‘nanocarriers’ offer a potential solution to the problems associated with conventional chemotherapy. Nanocarriers are drug delivery systems (DDSs) designed to deliver APIs to a cellular site of interest in a targeted manner (Peer et al., 2007; Bartkowski and Giordani, 2021). Many different types of nano-structures are commonly used as scaffolds to produce nanocarrier systems. Notable examples include liposomes, polymeric NPs, polymerosomes, micelles, dendrimers, and various carbon nanomaterials (Peer et al., 2007). Each type of scaffold has unique features and physiochemical characteristics, such as size, loading capacity, surface area, and colloidal stability; all of which afford their suitability for a specific application. To create a nanocarrier, these scaffolds may be functionalised (covalently or non-covalently) with different moieties, as to impart extended functionality.

Therapeutics encapsulated in a nanocarrier system may see their solubility, pharmacokinetic profile and half-life markedly improved. The nanocarrier my impart this improvement by: protecting the drug from degradation during distribution; encapsulating the drug to lower its systemic toxicity; enabling the drug to penetrate and absorb into desired tissues; releasing the drug at a desired site in a targeted manner; and allowing the drug to bypass the aforementioned MDR transporters (Peer et al., 2007). In fact, in key part to their benefits over conventional formulations, several nanocarrier systems have been clinically approved, with many more undergoing clinical trials (Weissig and Guzman-Villanueva, 2015; Bobo et al., 2016).

A typical nanocarrier system consists of a scaffold functionalised with a targeting moiety and a drug payload/cargo. The targeting moiety enables the system to be selectively uptaken by cancer cells. For instance, hyaluronic acid may be used to impart targetability towards cancer cells overexpressing the CD44 receptor (Dosio et al., 2016). The scaffolds are typically selected and optimised to release the drug payload when exposed to specific stimuli, which may result in their protonation, hydrolytic cleavage or supramolecular conformational change (Mura et al., 2013). However, a major limitation to scaffolds in nanocarriers is that they may be unresponsive to the specific stimuli required to induce drug release. Fortunately, stimuli-responsive control over drug release can be enabled in these systems by incorporating ‘molecular switches’.

A molecular switch can be termed a molecule that can be manipulated to transition from one state to another when exposed to electrical, chemical or optical stimuli (Raymo, 2002; Li and Qu, 2015). As a molecular switch absorbs the external energy of a particular stimulus it is designed to respond to, its electronic configuration is altered, resulting in a detectable signal. The types of transitions a molecular switch may undergo include *cis* → *trans*/*trans* → *cis* isomerisation, ring-flipping, ring-opening/ring-closing, and intramolecular proton-transfer processes (Natali and Giordani, 2012). Most notably, molecular switches come with the advantage of this transition being reversible and repeatable. Together, the properties of molecular switches have enabled their incorporation into responsive materials and optoelectronic devices, for decades (Li and Qu, 2015).

Recent advancements in nano-based drug delivery have enabled the incorporation of molecular switches into DDSs (Bulmus, 2005). Molecular switches offer a non-invasive and bio-compatible mechanism by which encapsulated bioactive compounds can be released at a target site in a responsive and controlled manner when a specific stimulus is applied. Hence, molecular switches can improve the selectivity and efficacy of the administered drug and enhance its biosafety profile (Raza et al., 2019; Ghani et al., 2021). The different stimuli that can trigger these molecular switches can generally be divided into endogenous and exogenous stimuli (Raza et al., 2019).

Endogenous stimuli, that is to say, internal stimuli/biological stimuli, can include different levels of pH, the presence of different reductive and oxidative chemical species, and the presence of different enzymes (Raza et al., 2019). In other words, physiological differences between the microenvironments of a disease area and healthy tissue often serve as endogenous stimuli. The changing biochemistry of fast-growing cancerous cells can trigger a response in molecular switches designed explicitly for changes in pH, redox response or enzyme activity. For pH sensitivity, the contrast in the pH of 5.5 in the cytoplasm of a tumorous cell compared to a pH of 7.4 in healthy cells can be exploited to activate molecular switches (Shen et al., 2015). Changes in pH can also be used in the cleavage of a linker holding a drug bound to a nanocarrier. An example is a hydrazone bond that is stable under neutral pH but cleaved in more acidic conditions (Yoo et al., 2002). Enzymes perform an essential part of many physiological functions, such as peptide bond cleavage and protein formation. These enzymes may exhibit upregulation in diseased microenvironments; thus, their heightened activity can be exploited. For instance, certain types of cancerous cells present elevated levels of glycosidases and proteases, which have been targeted for selective drug therapies (Hu et al., 2014). Redox homeostasis is critical to regular cellular activity. Redox dysregulation is a typical hallmark of cancerous cells, resulting in elevated levels of many reactive oxygen species (ROS) and increased antioxidant ability. The primary source of oxidative stress in cancer cells involves the radical ROS: hydroxyl radicals (^•^OH) and superoxide (O_2_^•-^); and the non-radical ROS: hydrogen peroxide (H_2_O_2_) and singlet oxygen (^1^O_2_) (Liou and Storz, 2010). In response to increased levels of ROS, cancer cells increase their antioxidant defences. For instance, the antioxidant glutathione (GSH) becomes upregulated (Panieri and Santoro, 2016). This elevation of ROS and anti oxidant species in cancer cells has led to the emergence of reduction-responsive drug carriers (Quinn et al., 2017).

Exogenous stimuli, that is, stimuli arising from outside of the body, can be advantageous as they can activate molecular switches remotely. Exogenous stimuli may include electric fields, ultrasound or light irradiation. Electric fields may be useful in DDSs that utilise conductive polymers. When exposed to a direct-current (DC) electrical field, polypyrrole-based nanoparticles have been shown to release their drug payload (Ge et al., 2012). Ultrasound has been shown to produce pores in blood vessel walls in a sonoporation process, permitting targeted delivery of cancer treatments to target sites with otherwise limited accessibility (Mullick Chowdhury et al., 2017). Aside from electrical and sound stimuli, the exogenous stimulus that has attracted the most interest in DDSs is light. This interest is due to the versatility and ease with which light is manipulated, eliciting a delicate control to its application (Linsley and Wu, 2017). Two categories of non-ionising light commonly utilised for drug delivery purposes are visible (VIS, ∼400–750 nm) and near-infrared (NIR, ∼750–2,500 nm) light (Tao et al., 2020). In certain approaches, ultraviolet A (UVA, 315–400 nm) light has also been applied in this regard (Karisma et al., 2021).

Of the different stimuli listed above, light and changes in pH are two of the most versatile and accessible triggers with regards to molecular switches. In this regard, the following three sections focus on DDSs that incorporate molecular switches to enable light and pH-responsive control over drug release.

## DDSs With Switches—Controlled by pH

Abnormal conditions, such as inflammation or hypoxia, are typical in cancerous tumours (Hanahan and Weinberg, 2011). These conditions lead to structural and physiological changes, atypical of healthy tissues. For instance, the endothelial lining of blood vessels becomes more permeable than normal, and lymphatic drainage is significantly reduced. Together, these two hallmarks of tumour tissues are known as the enhanced permeability and retention (EPR) effect (Torchilin, 2011). This EPR effect enables large molecules and NPs to accumulate inside the interstitial space (Torchilin, 2011). This accumulation is a form of passive targeting DDSs may utilise towards tumours. EPR-based delivery relies solely on the unique permeability of the tumorous vessel wall for nanocarrier accumulation. Studies of tumour permeability have demonstrated that the typical vascular cutoff threshold for vesicle size is ∼400–600 nm (Yuan et al., 1995; Hobbs et al., 1998). However, particles below 200 nm have shown to be most effective at extravasation into tumours (Peer et al., 2007). A limiting factor to this increased vessel permeability is that it may vary within the same tumour, and various tumours do not exhibit the EPR effect (Peer et al., 2007).

The targeting of tumour tissues through the EPR effect has undoubtedly benefited the therapeutic efficacy of nanocarrier-based delivery (Peer et al., 2007). However, as it is a form of passive targeting, issues arise in regards to controlling therapeutic release from the DDS. ‘Smart’ DDSs are more complex systems that take an active approach to drug delivery. Smart DDSs are designed to communicate with and respond to changes in the desired target site’s microenvironment, hence enabling targeted and controlled drug release (Alvarez-Lorenzo and Concheiro, 2013). Topically, incorporating molecular switches into DDSs may enable targeted and controlled drug release. In this regard, the use of molecular switches is a proven, but complex, approach to improving an APIs efficacy, safety profile, and systemic toxicity (Mohapatra et al., 2018). Once a DDS is delivered to a target area, the incorporated molecular switch may activate when exposed to particular stimuli. The resulting conformational change in the molecular switch will, thus, induce the release of the drug cargo from the DDS.

A stimulus that molecular switches are commonly designed to react to, and the topic of this section, is changes in pH. This endogenous stimulus is of particular interest for DDSs designed for anticancer therapies. The tumour microenvironment has characteristic site-specific acidic irregularities and an overall low pH, resulting from the ‘Warburg effect’ (Warburg, 1956). The Warburg effect describes a situation where the acidity around cancer cells is enhanced due to increased anaerobic glycolysis, which results in the formation of acidic lactate (Warburg, 1956; Iturrioz-Rodríguez et al., 2019).

Liposomes have been well studied for their application in drug delivery (Sharma, 1997; Lian and Ho, 2001). Briefly, liposomes are microparticulate vesicles characterised by a phospholipid bilayer. They form spontaneously when specific lipids are exposed to aqueous media. Their size range (∼20 nm–30 µm), general biocompatibility, ease of preparation, high drug loading capacity, and their ability to enter cells through different mechanisms has led to many clinically approved liposomal-based nanocarrier formulations (Sharma, 1997; Beltrán-Gracia et al., 2019). A further benefit of liposomes is that their lamella (lipid bilayer, of which can have multiple) may be modified to optimise site-specific targeting. For instance, covalent conjugation with poly(ethylene glycol) (PEG) has resulted in improved circulation times through reduced immunogenicity, and the addition of cationic lipids in the membrane has been shown to increase the ability of liposomes to infiltrate cells (Badkas et al., 2018; Zhao et al., 2019). Liposomal lamellar modification has also concerned the incorporation of molecular switches for stimuli-triggered drug release.

**FIGURE 1:**
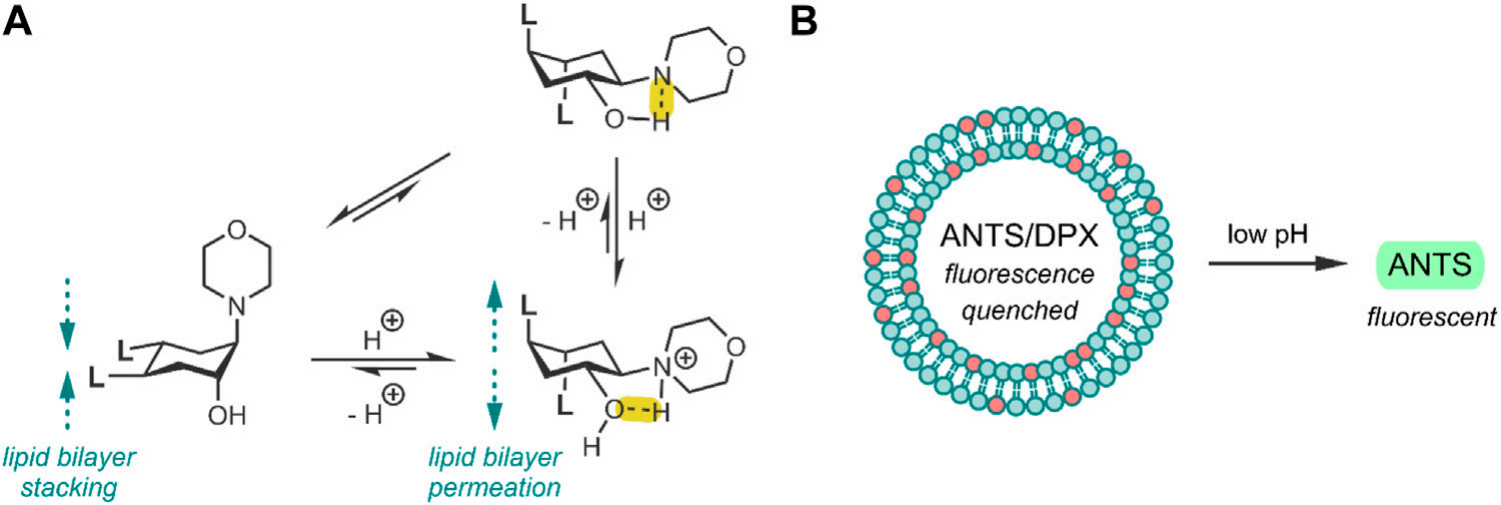
**(A)** pH-induced conformational change of the TACH-lipid derivative, where **L** is the lipid tail -COOC_12_H_25_. Hydrogen bonds have been highlighted in yellow. **(B)** 25 mol% TACH-lipid derivative (red) and 75 mol% POPC (blue) liposome formulation containing the ANTS/DPX dye/quencher pair. Acidic conditions result in liposomal leakage and the dequenching of ANTS (Brazdova et al., 2008).

In 2008, Brazdova et al. took the approach of using *trans*-2-aminocyclohexanol (TACH) molecular switch for their liposomal formulation, whereby the group derived TACH with lipid tail ends (Brazdova et al., 2008). The resulting amphiphilic TACH-lipid derivative was able to form liposomes in an aqueous solution. By exposing these liposomes to low pH, the group demonstrated that the protonation of the morpholine nitrogen of the TACH-lipid derivative drives the conversion to the alternate chair formation (Figure 1A). Intramolecular hydrogen bonding in the TACH-lipid derivative forces both the morpholine and hydroxyl groups into equatorial positions, resulting in the appended hydrophobic lipid chains to shift into their axial position. The group speculated that this shift could interfere with the standard liposome morphology and lipid packing, hence triggering the release of liposomal content (Brazdova et al., 2008).

The group then investigated their TACH-lipid derivative’s triggered liposomal content release properties. For this, liposomes containing 25 mol% TACH-lipid derivative and 75 mol% 1-palmitoyl-2-oleoyl-*syn*-glycero-3-phosphocholine (POPC) were prepared. POPC was added as a co-lipid as it assists in forming lamellar structures (Duelund et al., 2013). A 8-aminonaphthalene-1,3,6-trisulfonic acid (ANTS)/*p*-xylenebis-pyridinium bromide (DPX) dye/quencher pair (ANTS/DPX) (Table 2), were used as model cargo. ANTS/DPX are commonly used to assay liposomal leakage and lipid-mixing. The assay operates on the premise that DPX quenches ANTS when inside the liposome. When ANTS leaks out, it becomes dequenched, and its fluorescence can be observed (Lins et al., 2002; Guo et al., 2003). The TACH-lipid derivative: POPC liposomes were stable for over 1 h at 7.4 pH and 37°C, with no substantial ANTS leakage. However, when exposed to pH 5.5, an ANTS release of ∼50% was observed after 10 min (Figure 1B). The group also investigated another TACH-lipid derivative in different liposomal formulations. Similar functionality was observed in these systems, with mildly acidic conditions resulting in the release of ANTS. Overall, Brazdova et al. highlight the potential of the amphiphilic TACH-lipid derivatives for pH-responsive liposomal delivery of drugs and genes (Brazdova et al., 2008).

Following the research of Brazdova et al., the idea of incorporating a switch into the lamellar structure of liposomes was further investigated by (Viricel et al., 2015). In their study, the group demonstrated the use of alkyl-chain functionalised molecular switches to deliver and release drugs, which are typically cell-impermeable, into the cytosol. The group asserted that their system improves endosomal escape, which is a significant challenge for liposomes entering the cell through an endocytosis pathway (Viricel et al., 2015). Specifically, endosomal escape of the liposome has to be realised in under an hour, which is the approximate timescale of endosomal maturation. Otherwise, the liposomes may be delivered to the lysosome, where they will be degraded (Huotari and Helenius, 2011; Scott et al., 2014).

**FIGURE 2:**
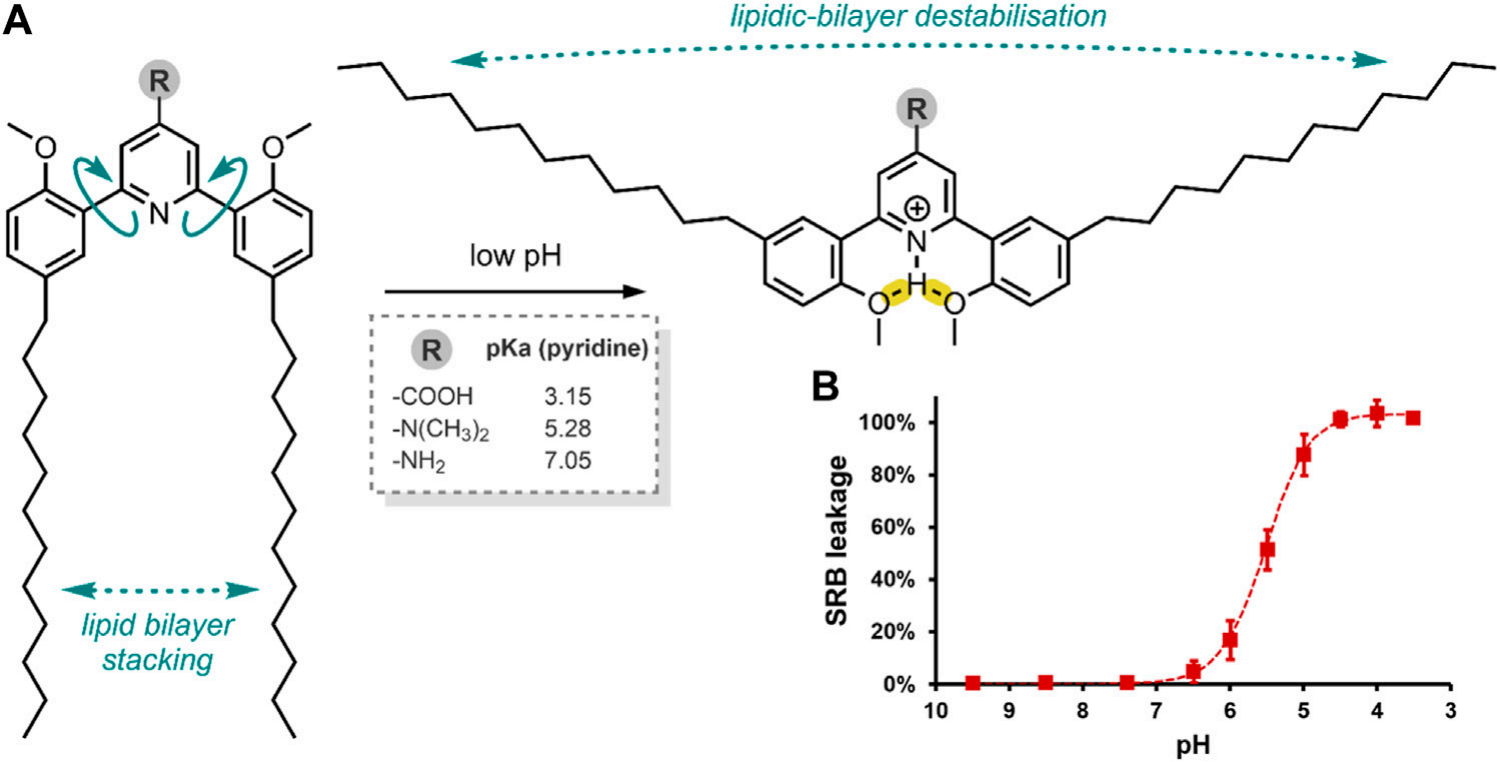
**(A)** pH-induced conformational change of the alkyl-chain functionalised di(methoxyphenyl)-pyridine molecular switch, resulting in liposome destabilisation and SRB release. *Framed*: the effect of the R headgroup on pyridine pKa. Hydrogen bonds have been highlighted in yellow (Viricel et al., 2015). **(B)** SRB leakage from a liposome formulation containing the switch with the –N(CH_3_)_2_ headgroups (75 mol%), at decreasing pH after 15 min.

Viricel et al. used di(methoxyphenyl)-pyridine as the basis of a pH-responsive molecular switch, which they derivatised with two alkyl chains (Figure 2A). To enable a switching capability, a polar headgroup was required to be introduced to the pyridine at the para position. The group prepared three such derivatives of the switch-alkyl conjugate, using three different polar headgroups to optimise the pH-responsive range of their liposomes. Through *in silico* approximations, the group identified three headgroups covering a range of pKa (pyridine) values (Figure 2A). Having prepared their three switches, the group examined their switching capability when exposed to acidic pH. The switching capability was confirmed by NOESY NMR and a ^1^H NMR titration, and it was suggested that the switching mechanism involves a rotation along the C_pyr_—C_phe_ bond (Viricel et al., 2015).

The Viricel et al. group then prepared various liposome formulations using their switch conjugates at 25, 50 or 75 mol%, with the co-lipids 1,2-distearoyl-*sn*-glycero-3-phosphocholine (DSPC) and 1,2-distearoyl-*sn*-glycero-3-phosphorylethanolamine-*N*-[amino (polyethylene glycol)-2000] (DSPE-PEG_2000_), and the incorporation of sulforhodamine B (SRB) (Table 2) as a model of a highly polar payload. The co-lipids, DSPC and DSPE-PEG_2000_ were added to aid liposomal formation. SRB was chosen as this dye cannot cross endosomal and cytoplasmic membranes on its own, and it can be visualised through fluorescence microscopy (Vichai and Kirtikara, 2006). Hence, SRB enabled the group to assess their liposome formulations’ cellular colocalisation and endosomal escape. Of the three headgroup-switch-alkyl conjugates, only those with the –COOH and –N(CH_3_)_2_ headgroups formed liposomes. The conjugate with the –NH_2_ headgroup could not form a liposome as the pKa (pyridine) was too high. The 25, 50 or 75 mol% formulations with the –N(CH_3_)_2_ headgroup showed excellent pH-responsive SRB release, with the 75 mol% formulation achieving complete release of SRB after 5 min at pH 4.5 (Figure 2B) (Viricel et al., 2015)**.**

Systems delivering a fluorescent cargo allow for understanding where the release occurs and the elucidation of the cell internalisation pathway (Bartelmess et al., 2015). The Viricel et al. group incubated the 50 mol% formulation with the –N(CH_3_)_2_ headgroup in HeLa cells to assess its ability to deliver SRB to the cytosol. Indeed, the group demonstrated the successful cellular delivery and endosomal escape of the highly polar SRB through fluorescence microscopy. The delivery system enabled fast and efficient endosomal drug-cargo escape, with an 88% release observed within 15 min at pH 5. Overall, the group developed a rapidly dissolving liposome at low pH, stable for several months at pH 7.4 (Viricel et al., 2015).

In a follow-up study, Viricel et al. furthered the application of their alkyl-chain functionalised di(methoxyphenyl)-pyridine molecular switch (Figure 2A) through further optimisation of the polar headgroup and the final liposomal formulations (Viricel et al., 2017). In this study, the group opted to deliver small interfering RNAs (siRNA) to HeLa cells. siRNAs are short (∼22 nt) duplex strands of RNA comprised of a sense/passenger strand and an antisense/guide strand (Table 2) (Alshaer et al., 2021). siRNA can be used for therapeutic purposes of many diseases due to its ability to regulate gene expression by interfering with the normal replication process of RNA (Bramsen and Kjems, 2012; Fakhr et al., 2016). In summary, the group demonstrated that their optimised liposomes efficiently deliver the therapeutic cargo with a pH-triggered release (Viricel et al., 2017). Viricel et al. conclude that the unique properties of stimuli-responsive molecular switches embedded in lipidoid material could innovate the field of gene and drug delivery (Viricel et al., 2017).

In summary, pH as a stimulus for molecular switch activation is a dependable trigger, as different physiological target sites, especially tumorous ones, have very characteristic pH environments (Warburg, 1956). A molecular switch may be optimised to respond to a specific pH, as has been demonstrated by Viricel et al. (Viricel et al., 2015, 2017). When a pH-responsive switch is incorporated into a DDS, it enables the system to release its drug cargo when delivered to a target site with an acidic character. The following section will focus on delivery systems incorporating molecular switches for light-responsive drug release. In particular, case examples are discussed where UV, VIS and NIR light are used as stimuli.

## DDSs With Switches—Controlled by Light

Of the different exogenous stimuli utilised in DDSs, light has been the most suited for drug release due to its precise spatiotemporal control and negligible interference with standard biochemical processes (Tao et al., 2020; Welleman et al., 2020). Light irradiation can be remotely applied, and its wavelength, intensity, and exposure duration can be adjusted to suit specific needs, resulting in high levels of pharmacological control (Aleandri et al., 2015).

Molecular switches that are responsive to light are sometimes called photochromics. Photochromic behaviour is where a species is interconvertible between two isomers on exposure to specific wavelengths of light and where each isomer has a different absorption spectrum. Many such photochromic materials have been reported across chemistry, materials science, physics and engineering, with interest rapidly rising since the 1990s (De Sousa et al., 2021). This section will focus on cases studies of molecular switches with photochromic behaviour, which have been used to impart light-responsive control of drug release on DDSs.

The structural changes in a molecular switch accompanied by the absorption of light energy may manifest as a ring-opening/closing or a *cis*/*trans* isomerisation. As a photochromic molecule absorbs light energy, the resulting higher energy isomer is less stable. Generally, this isomer can reverse spontaneously/thermally to its more stable state if not stabilised. Alternatively, its reversion may be induced by applying another stimulus, such as hear or a lower-energy wavelength of light. The most critical photochromic molecular switch families include spiropyrans (SPs), fulgides, dithienylethenes, dihydroindolizines, chromenes, and azobenzenes (Natali and Giordani, 2012). In this section, azobenzenes and SPs are discussed for their ability to impart light-responsive control for drug delivery.

**FIGURE 3:**
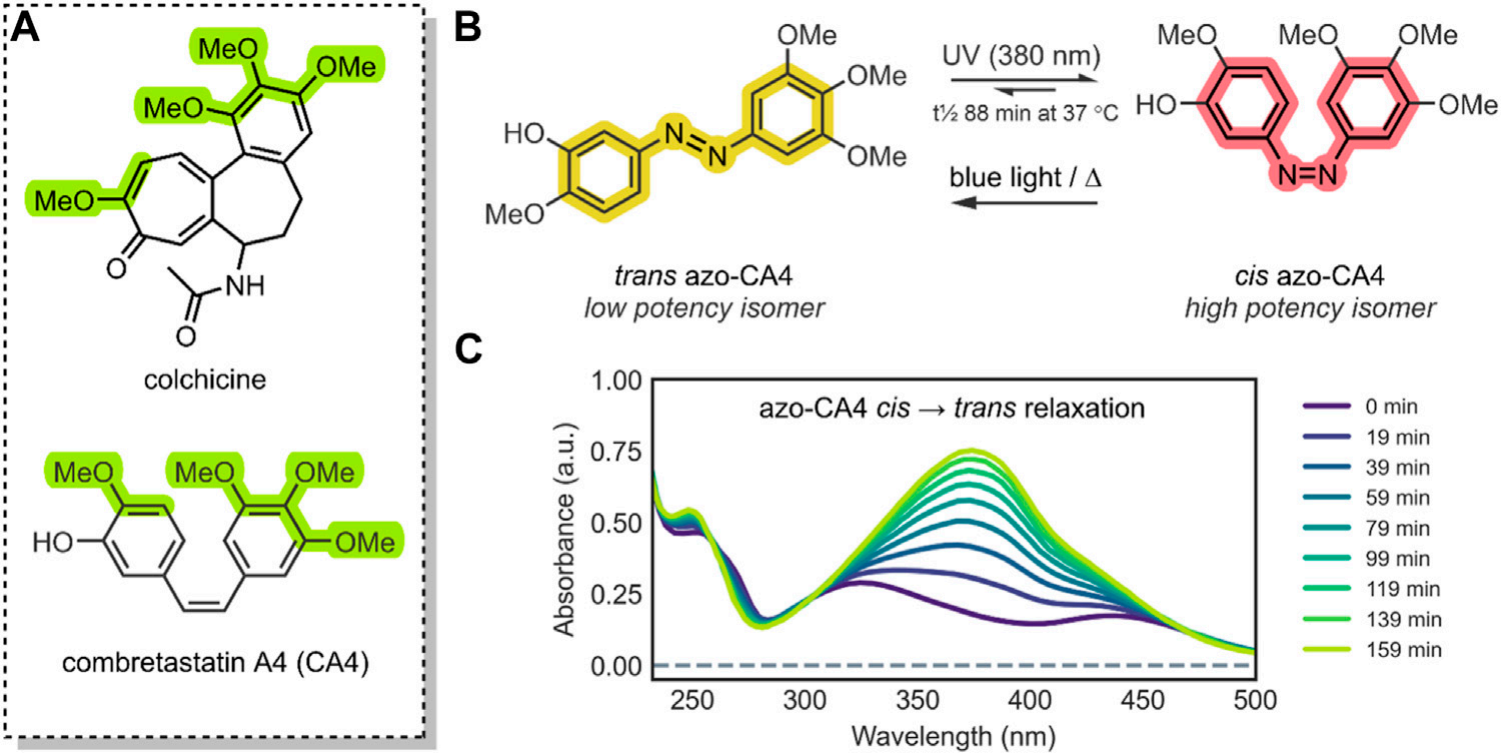
**(A)** The structure of CA4 (antagonist of the colchicine-receptor of tubulin) and colchicine; the pharmacophore of CA4 has been highlighted green (Borowiak et al., 2015). The azobenzene core has been highlighted; the colours, yellow (*trans* azobenzene) and red (*cis* azobenzene), are roughly representative of the compounds’ physical colour (Hofmann et al., 2012; Norikane et al., 2016). **(B)** The isomerisation of *trans* azo-CA4 to *cis* azo-CA4 (Sheldon et al., 2016). The *cis* azo-CA4 mimics the structure of CA4 (Mulatihan et al., 2020). **(C)** UV-VIS absorption spectra of azo-CA4 in DMSO, dark and 37°C, after irradiation (380 nm, 1 min, 4.4 mW/cm^2^); thermal relaxation of *cis* azo-CA4 (λ_max_ ∼325 & 440 nm) to *trans* azo-CA4 (λ_max_ 379 nm) is evident, with a t½ of ∼88 min. This UV-VIS absorption spectrum is similar to other azobenzene species (Rau and Lueddecke, 1982; Poutanen et al., 2016).

Azobenzenes are structurally the simplest of the aforementioned families; simply, they are molecules with a core of two phenyl rings coupled through an N=N double bond (Figure 3B). Each phenyl ring can be functionalised at its ortho, meta and para positions. Azobenzenes are also commonly referred to as diazastilbenes because of their similarity to the stilbene core. Azobenzenes have two isomeric forms: the *E* (*trans -*N=N-) isomer and the *Z* (*cis -*N=N-) isomer. When in their *trans* form, azobenzenes are stable and planar. As molecular switches, *trans* azobenzenes are responsive to UV light (∼360–380 nm), which results in their reversible conversion to the *cis* isomer (Rau and Lueddecke, 1982; Natali and Giordani, 2012). The specific absorption λ_max_ is dependent on its phenyl substituents, as well as solvatochromic and acidochromic effects (Hofmann et al., 2012). This direct *trans* → *cis* isomerisation process occurs through a π-π* electronic transition. The resulting *cis* form of azobenzene is bent/folded and less stable. The reverse *cis* → *trans* back-conversion happens thermally over time, and it can be accelerated by VIS light irradiation or the application of heat, or in certain cases, it can be catalysed through redox chemistry (Samanta et al., 2013). The *cis* → *trans* reversion occurs through an n-π* electronic transition. This simple isomerisation process of azobenzene results in a high quantum yield. The two isomers of azobenzene (*E* & *Z*) also differ in their properties, including dipole moment, colour and conformation (Cardano et al., 2018). The simplicity of the isomerisation mechanism, differing properties of each form, accessible and modable structure, ease of reversibility, and high quantum yield, afford azobenzenes suitability to applications requiring rapid and reversible switching behaviour. Furthermore, the molecules are versatile as their switching can be modified through the substituents on the phenyl rings. Depending on their position on the ring and whether they are electron-donating or withdrawing, substituents can offer an element of control that can help tune the switch for its specific function (Merino, 2011; Bandara and Burdette, 2012). Together, these properties position azobenzenes as highly suitable molecular switches for drug delivery applications.

In all cases discussed to this point, a conformational change of the molecular switch is used to release a drug payload by distorting the structure of its carrier. An attractive, atypical approach to minimise toxicity and focus the APIs potency to the tumorous site is to incorporate the photochromic mechanism into the structure of the therapeutic API itself, thus mimicking the drug with a photochromic agent (Riefolo et al., 2021). A method employed by Sheldon et al. was to use the core of an azobenzene to engineer a photochromic analogue of combretastatin A-4 (CA4) (Sheldon et al., 2016).

Combretastatins are a natural class of products originating from the *Combretum caffrum* African Bushwillow and are characterised by their phenolic-stilbene core (Nainwal et al., 2019). Combretastatins are potent inhibitors of angiogenesis and cellular proliferation (Sherbet, 2020). Their mechanism of action involves binding to the colchicine pocket of the tubulin protein, thus inhibiting its polymerisation into microtubules, without which normal cellular dynamics are not possible (Seddigi et al., 2017). The specific combretastatin selected by Sheldon et al. for their study, CA4, is one of the most effective combretastatins for its superior antiproliferative properties; it has been used to treat solid tumours and retinal neovascularization (Seddigi et al., 2017; Nainwal et al., 2019). The CA4 core contains a cis-stilbene; in this orientation, the molecule mimics colchicine (Figure 3A), resulting in <10 nM EC_50_ anticancer potency. However, the trans isomer of CA4 is 60-fold less potent (Sheldon et al., 2016). As such, in principle, by reversibly controlling the *cis*/*trans* isomerisation of CA4, its potency could be turned ON/OFF.

To impart this switching behaviour on CA4, the group replaced the stilbenoid core with an azobenzene core (azo-CA4) (Figure 3B). Through *in silico* studies, the group confirmed that their azo-CA4 analogue, when in the *cis* form, is highly similar in conformation to CA4, and therefore, should bind to the colchicine receptor. The introduction of the azobenzene core, with its N=N bond, enabled rapid and reversible switching control, which was previously impossible with the C=C bond of CA4. When exposed to UV light (380 nm) irradiation, the molecule would isomerise from *trans* azo-CA4 to the *cis* azo-CA4 drug-mimicking analogue. However, the *cis* azo-CA4 was unstable and would revert to the thermodynamically favourable *trans* isomer over time (Figure 3C). With 1 min of UV light irradiation, the absorption shifted to the profile for *cis* azobenzene, which reverted with a half-life (t½) of 75–100 min. More extensive thermal relaxation studies revealed the specific t½ to be 88 min. As such, for bio-purposes, azo-CA4 will require a UV-light pulse regimen for best therapeutic effects (Sheldon et al., 2016).

A further issue associated with azobenzenes for biomedical applications is that they are known to be reduced by GSH. The attack of GSH on the diazo nitrogen may result in *cis* → *trans* relaxation or the molecules’ destruction. Through a GSH reduction assay and LC-MS, the group demonstrated that, unfortunately, the latter occurs with their azo-CA4. This degradation poses issues with high-dose administration of azo-CA4 to GSH upregulated cells. However, it is possible to impart resistance to reduction by GSH by replacing the azo-CA4 methoxy substituents with more electron-withdrawing groups (Samanta et al., 2013).

The group then investigated the biological efficacy of their azo-CA4 photochromic drug. *In vitro* cell viability assays were carried out against the human umbilical vein endothelial cell line (HUVEC). Expectedly, the azo-CA4 did not have the low nM potency characteristic of CA4. This was justified by the aforementioned presence of GSH, which in contrast, does not act on CA4. Regardless, the *cis* azo-CA4 showed good results. When azo-CA4 was administered to HUVEC cells and pulse-irradiated with UV light (1 min every 1 h for 48 h, 380 nm, 4.4 mW/cm^2^), an EC_50_ of ∼400 nM was observed. Expectedly, the azo-CA4 had significantly lower activity without irradiation, with an EC_50_ of ∼9.5 µM; a 22-fold decrease. For comparison, the group administered CA4 to the HUVEC cells, which resulted in an EC_50_ of ∼2.1 nM. Further biological studies involved *in vitro* tubulin polymerisation assays. These assays showed that, by irradiating azo-CA4 (1 min, 380 nm, 4.4 mW/cm^2^), its tubulin-inhibition potency increased 2.8-fold (Sheldon et al., 2016).

In conclusion, incorporating a molecular switch’ core into an API has been shown to enable reversible control over its therapeutic activity (Sheldon et al., 2016). The delivery of a photochromic API in its OFF state to a target site, and the fast photo-isomerisation to its ON state, can enable tight control of therapeutic potency and limit its activity at non-target sites. *Ipso facto*, a molecular switch can impart exogenously controlled prodrug-type functionality on an API.

Sheldon et al. successfully used UV light to activate their drug analogue in *vitro* experiments (Sheldon et al., 2016). However, it should be noted that UV-light irradiation may not be an ideal choice for biomedical purposes. Exposure to UV light under 300 nm is harmful at the cellular level as it generates photoreactions in nucleic acids and proteins (Gonzaga, 2009; Figueiras et al., 2011). Furthermore, UV light may have limited application *in vivo* as it has very low tissue penetration due to absorbance and scattering by lipids and water (Liu et al., 2016).

More recently, attention has turned to develop DDSs responsive to NIR light. There are four tissue transparency ‘optical windows’ considered in biological applications of NIR light. These are, NIR-I (∼700–1,000 nm), NIR-II (∼1,000–1,350 nm), NIR-III (∼1,550–1870 nm), and NIR-IV (∼2,100–2,300 nm) (Golovynskyi et al., 2018). To note, the NIR-III and NIR-IV optical windows overlap with the short-wave infrared (SWIR) range of light (∼1,400–3,000 nm). In fact, NIR-III is occasionally referred to as the SWIR-I window, and NIR-IV is frequently referred to as the SWIR-II window (Golovynskyi et al., 2018). These four optical windows offer tissue penetration of ∼3 cm, thus offering a solution to *in vivo* chemotherapeutic applications by effectively decreasing cellular damage (Jalani et al., 2016; Yang et al., 2017). However, the exact penetration depth may vary with wavelength, intensity and type of tissue irradiated. For instance, the NIR-II and NIR-III windows have the highest tissue permeability in all tissue types, whilst all four NIR optical windows have decreased permeability in bone tissue (Golovynskyi et al., 2018).

**FIGURE 4:**
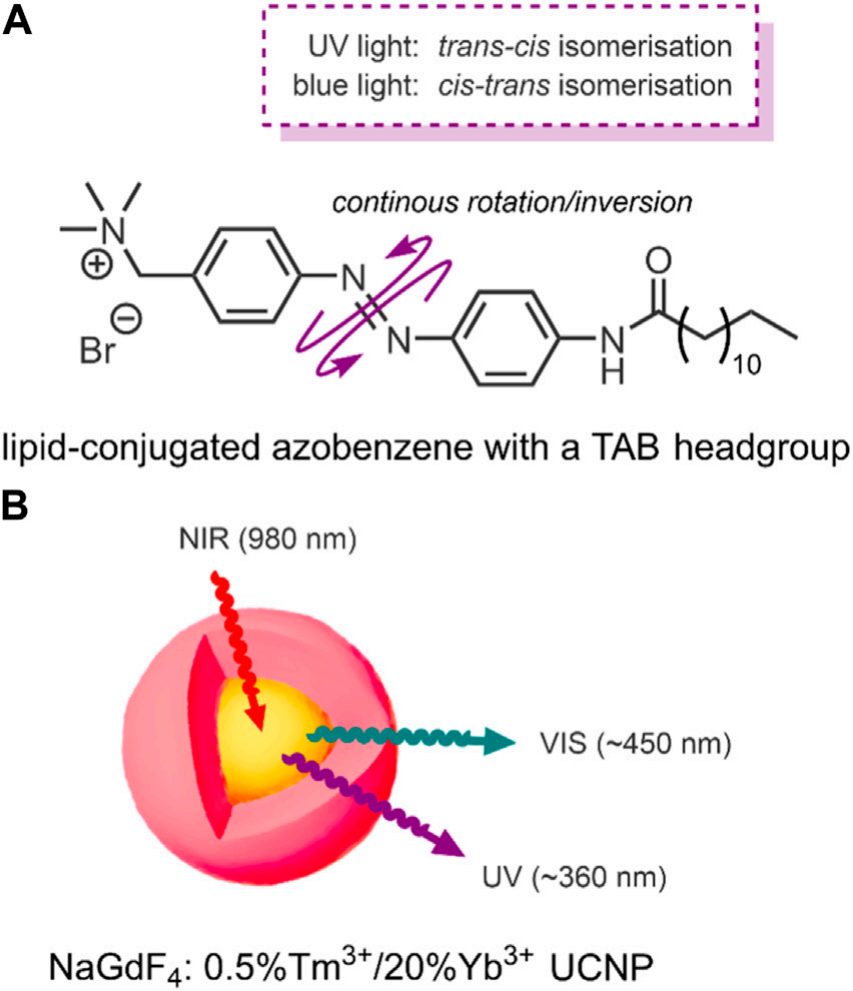
**(A)** Amphiphilic lipid-conjugated azobenzene with a TAB headgroup. The switch undergoes a continuous rotation/inversion on irradiation with UV and VIS light (Yao et al., 2016). **(B)** NaGdF_4_: 0.5%Tm^3+^/20%Yb^3+^ core-shell (highlighted yellow-red) UCNPs upconvert 980 nm NIR light into ∼360 nm UV and ∼450 nm VIS light (Yao et al., 2016).

Regarding photochromic applications, NIR light may not have the energy required to trigger a photoswitch to isomerise. Many molecular switches require irradiation with the UV range of light for isomerisation (Natali and Giordani, 2012). A solution to this issue is a process called upconversion, whereby two or more low-energy photons (long-wavelength) are combined to generate a single high-energy photon (short-wavelength) in a nonlinear optical phenomenon (Luan et al., 2021). This process is known as the anti-Stokes effect (Li et al., 2015). Research from the last decade has focused on developing composite nanomaterials termed upconversion nanoparticles (UCNPs) (Chen et al., 2014). These UCNPs enable NIR conversion into higher energy UV/VIS light (Figure 4A), which is required to produce the requisite conformational in certain molecular switches (Bettinelli et al., 2015; Lee and Park, 2018).

Conventional NIR-triggered liposomal DDSs convert light energy into heat by incorporating photothermal NPs, such as hollow gold nanoshells (Wu et al., 2008). The resulting temperature rise in these systems induces a phase transition between the phospholipids and the photothermal NPs, resulting in structural breakdown and payload release (Wu et al., 2008). In 2016, Yao et al. reported a NIR-triggered liposomal DDS, with the novel exception that their system used a molecular switch to impart NIR-responsive drug release control (Yao et al., 2016). The specific molecular switch used by the group was an amphiphilic lipid-conjugated azobenzene with a trimethyl ammonium bromide (TAB) headgroup (Figure 4A). However, azobenzenes absorb UV light; NIR light does not have sufficient energy to trigger the molecular switch. For this reason, Yao et al. employed the use of the aforementioned upconversion process by incorporating UCNPs (Yao et al., 2016).

The liposomes formed by the group were multilamellar structures (∼200 nm in size). The lipid bilayer was composed of their lipid conjugated molecular switch and the DSPC and dioleoyl-3-trimethylammonium propane (DOTAP). DSPC and DOTAP enhanced cellular uptake and minimised undesired cellular interactions. Within the lamella, the UCNPs and doxorubicin (DOX) were encapsulated (Yao et al., 2016).

DOX has an anthraquinone structure (Table 2) and is a highly popular antineoplastic agent as it targets a broad range of indications. DOX is effective against severally metastatic cancers, including, but not limited to, ovarian cancer, breast cancer, lymphoma, acute lymphocytic leukaemia, and Kaposi’s sarcoma (Peer et al., 2007). DOX is an intercalating agent, where its anthraquinone rings insert between the two strands of the DNA helix. In this position, DOX inhibits the replication and transcription function of the topoisomerase II complex, resulting in cell death (Upadhyay et al., 2021). Colloquially, DOX is defamed as the ‘Red Devil’ because of its vivid red colour and its harsh side effects; including, antibiotic activity, nephrotoxicity, myelosuppression extravasation, and cumulative cardiotoxicity (Chhikara et al., 2011).

The specific UCNPs used by Yao et al. consisted of a structured NaGdF_4_ core-shell, the NIR triggered component, which incorporates luminescent lanthanide ions. NaGdF_4_ is used because of its ability to mediate energy exchanges, as it has high photochemical stability and low vibrational energy (Czerny et al., 2020). This core-shell structure was doped with the luminescent lanthanides, thulium and ytterbium (NaGdF_4_: 0.5%Tm^3+^/20%Yb^3+^). These UCNPs upconvert NIR light (980 nm) into the UV/VIS region. When the group applied laser irradiation of 980 nm to their UCNP, two distinct emission bands were detected; UV light at ∼360 nm and blue VIS light at ∼450 nm (Figure 4B) (Yao et al., 2016).

To test the ability of their DDS for NIR-controlled drug release, the group used a dialysis device to simulate blood vessels. The device consisted of a dialysis membrane (blood vessel wall), in which their liposomes were placed. Outside of the membrane was a buffer solution. The group then irradiated their liposomal DDS with intermittent pulses of NIR light (980 nm, 2.2 W/cm^2^, 6 h). As the system was NIR-irradiated, the UCNPs produced UV/VIS light. The azobenzene switch then absorbed this UV/VIS light, resulting in repeated isomerisations was a continuous rotation-inversion movement (Figure 4A) within the liposome membrane. The combined steric effect of the *cis*-form and polarity change was enough to destabilise the lamella, resulting in DOX release. Over the 6 h of NIR-irradiation, a 57% DOX release was observed. The release increased to 90% over the same period when irradiated at 7.8 W/cm^2^. Furthermore, a stepped release profile was observed when irradiated in intermittent periods of 30 min, thus demonstrating precise release control. Under the same conditions, DOX-loaded liposomes formulated omitting the UCNPs were seen to release under 10% of their payload (Yao et al., 2016).

The group carried out *in vitro* studies on HeLa cells to confirm the drug-loaded liposomes’ cellular uptake and their cytotoxicity. These experiments indicated an endosomal internalisation pathway of their liposomal DDS. Without NIR light irradiation, their system showed good biocompatibility. Consequently, on NIR light irradiation (980 nm, 2.2 W/cm^2^), a correlating decrease in viability of the cancer cells with increased exposure times was observed. After 10 min of irradiation, cell viability decreased to ∼50% (Yao et al., 2016).

Overall, Yao et al. demonstrated that incorporating an azobenzene-based molecular switch, in conjunction with UCNPs, enables a DDS to perform a controlled and repeatable ON/OFF drug release under NIR light irradiation (Yao et al., 2016). The successful use of NIR light for controlling drug release is a biologically friendly approach because of its biosafety and good tissue penetration, as discussed earlier. Another family of molecular switches capable of imparting light-responsive control over drug release in DDSs are SPs.

**FIGURE 5:**
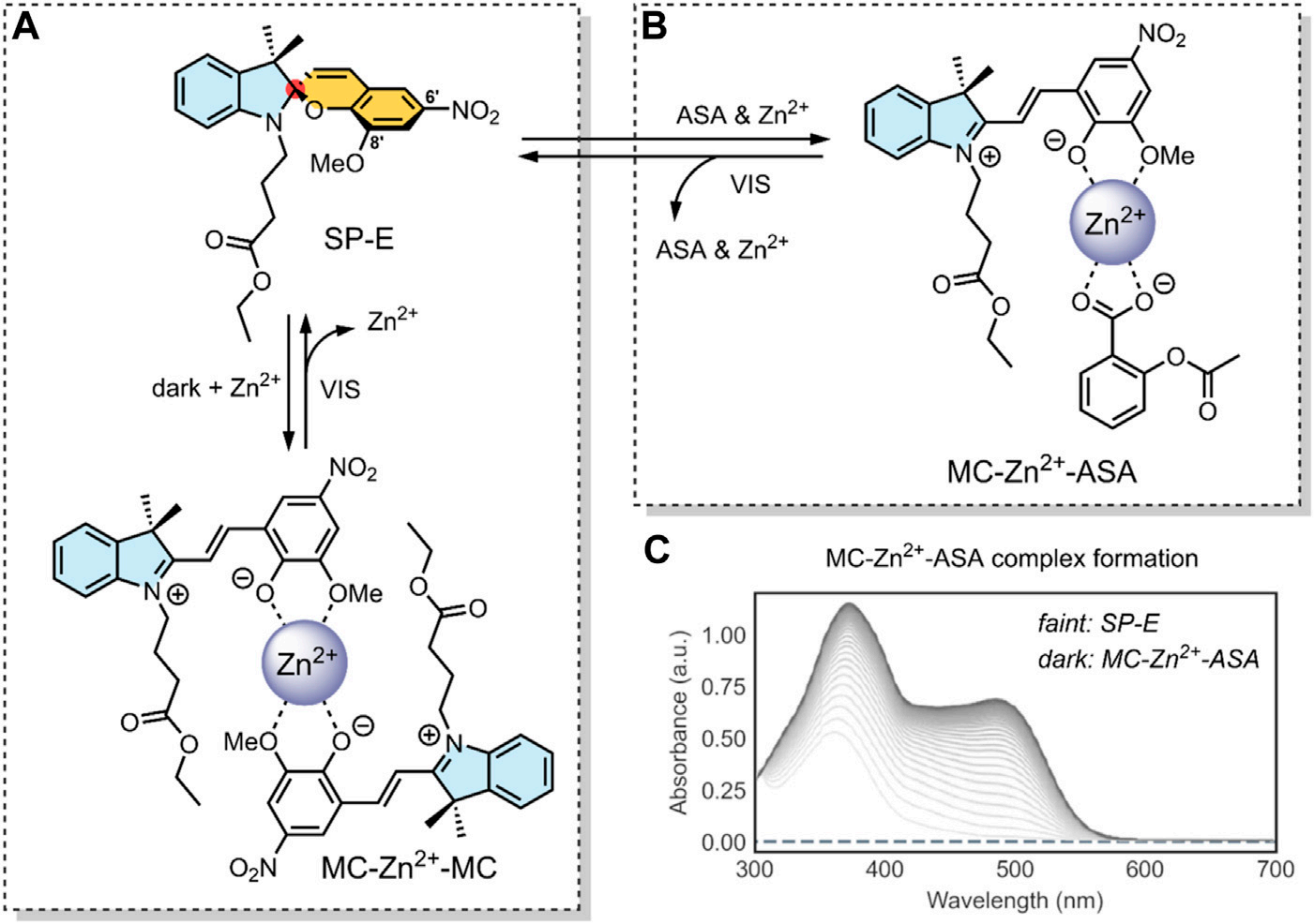
**(A)** Schematic of our SP-derivative, SP-E, with its 6′ nitro and 8′ methoxy groups; and the MC-Zn^2+^-MC complex, where MC is shown to chelate with Zn^2+^ through its phenolate and methoxy oxygens (Baldrighi et al., 2016). The spiro-carbon, indoline heterocycle, and benzopyran have been highlighted in red, blue, and yellow. **(B)** Schematic of the ternary supramolecular DDS, MC-Zn^2+^-ASA, designed by our group for reversible VIS light-triggered dual API release (Cardano et al., 2019). **(C)** UV-VIS absorption kinetics study of the MC-Zn^2+^-ASA ternary complex reformation (*faint grey to dark grey*) after a VIS-induced API release into solution, as measured in acetonitrile over 3 h immediately after irradiation, in the dark. The band at 490 nm is the λ_max_ of MC in MC-Zn^2+^-ASA; SP-E does not absorb in this wavelength (Cardano et al., 2019). *Figure C. adapted with permission from the Royal Society of Chemistry* (Cardano et al., 2019).

SPs are a popular example of molecular switches with photochromic behaviour (Machado et al., 2019). SPs have two main forms; the more-stable SP form and the less-stable merocyanine (MC) form. Each form has several distinct differences and properties, which enable the use of SPs for varied applications (Lukyanov and Lukyanova, 2005). The SP form consists of an indoline heterocycle perpendicularly joined to a benzopyran through a tetrahedral spiro-carbon atom (Figure 5A) (Natali and Giordani, 2012). SP is a hydrophobic, colourless, closed-form molecule with absorbance in the UV range between 200 and 400 nm. When UV light is absorbed, the carbon-oxygen bond of the tetragonal carbon is cleaved, resulting in SP → MC isomerisation. MC is an open-form planar molecule characterised by absorbance in the VIS region (Raymo and Giordani, 2001). MC is zwitterionic, and so it is hydrophilic. MC can isomerise back to the SP form in the presence of VIS light or through spontaneous relaxation over time. This easily-accessible reversible nature of the SP highlights its viability as a reversible photochromic switch for drug-delivery purposes (Cardano et al., 2019; Fagan et al., 2021).

In 2016, our group developed an SP derivative, termed SP-E, specifically designed to chelate with metal cations (Baldrighi et al., 2016). The SP-E derivative consisted of an *N*-modified 8-methoxy-6-nitrospiropyran. We tested the ability of this SP-E molecular switch to chelate with different (2+) metal cations. To this end, introduced the SP-E derivative to Mg^2+^, Zn^2+^ and Cu^2+^ cations. Results indicated that, when in the presence of a (2+) cation, the SP-E switch undergoes a metal-induced isomerisation into the MC form. Then, when in the MC form, the switch chelates the metal ion through the two oxygen atoms on its phenolate and methoxy moiety (Figure 5A). We identified that Zn^2+^ was an ideal metal cation for stabilising the switch in its MC isomeric form, resulting in an MC-Zn^2+^-MC complex (Figure 5A). This formation was confirmed as the MC-Zn^2+^-MC complex was responsive to VIS light, exhibited fluorescence, and had a hypsochromically shifted absorption band with respect to pure SP-E (Baldrighi et al., 2016).

In a 2019 follow-up study, we focused on using our SP-E molecular in a DDS for VIS light-controlled dual API release (Cardano et al., 2019). The first API was the metal cation Zn^2+^, which we have shown to chelate with our molecular switch. As the second API, we specifically use acetylsalicylic acid (ASA) (Table 2) as previous studies have shown that Zn^2+^ can form a complex with ASA (Lemoine et al., 2004; Cardano et al., 2019).

ASA, commonly known as aspirin, is a small-molecule drug that sees globally prevalent usage due to its analgesic, anti-inflammatory and antipyretic properties (Michalska-Małecka et al., 2016). ASA is a member of the nonsteroidal anti-inflammatory drugs (NSAIDs) class, which inhibit the cyclooxygenase (COX) enzyme. Specifically, ASA is known to inhibit prostaglandin H2 production by acetylating a residue in the active site of the COX-1 isoenzyme (Tóth et al., 2013).

In our 2019 study, we developed a ternary system through a supramolecular assembly. Our system combined a Zn^2+^ cation, the SP-E molecular switch, and ASA, resulting in an MC-Zn^2+^-ASA ternary supramolecular complex (Figure 5B). The formation of this ternary DDS was evidenced by UV-VIS absorption spectroscopy, which indicated a 1 : 1: 1 ratio of the MC: ASA: Zn^2+^. Through further titration experiments, we showed that the three components do not interact prior to assembly and that SP-E → MC isomerisation, induced by Zn^2+^ chelation, determines the rate of complex formation (Cardano et al., 2019).

The advantage of this molecular assembly is that the SP-E molecule switch is stabilised in its MC isomeric form, as SP-E cannot chelate with the Zn^2+^. Hence, when irradiated with VIS light (broad range LED), the induced MC → SP-E isomerisation results in the system’s collapse and ASA release. The complex would break down after irradiating with VIS light, resulting in ASA and Zn^2+^ dual release. UV-VIS absorption spectroscopy kinetic studies showed that the MC-Zn^2+^-ASA ternary complex would reform over 3 h, when in the dark (Figure 5C) (Cardano et al., 2019).

Overall, our study demonstrated the use of a spiropyran-based photochromic switch to impart VIS control over API release in a dual API delivery system. The cynosure of our DDS was that it was a ternary assembly comprised of only the two APIs and the molecular switch itself. Furthermore, the complex was formed through non-covalent interactions (Cardano et al., 2019). Together, these are favourable factors for large-scale DDS production; large scale production is a significant challenge associated with NP-based DDSs (Wilhelm et al., 2016).

Unlike DOX and ASA, which are readily solubilised in water, other drugs often suffer from decreased solubility. As discussed earlier, this is a significant challenge associated with many anticancer therapeutics. Poor solubility is especially the case in larger drugs molecules, where the introduction of solubilising bioisosteres does not significantly affect the drugs partition coefficient (Wermuth and Lesuisse, 2015). A notorious example of an anticancer drug with poor water solubility is paclitaxel (PTX) (Table 2); which has a water solubility of ∼0.5 μg/ml (Liu et al., 2011).

PTX is an antineoplastic agent employed to treat ovarian, breast and lung cancer. PTX is a member of the taxanes class, which function by inducing various cellular processes leading to apoptosis. The most prominent of which is promoting the polymerisation of the tubulin protein. The resulting microtubules are too stable to support normal cellular function, leading to cell death (Markman and Mekhail, 2002). This mechanism of action contrasts the tubulin polymerisation inhibition induced by CA4, as discussed earlier. Beyond the poor water solubility of PTX, the drug also suffers from a high cellular efflux (Liu et al., 2011). Due to these shortcomings, PTX is presently formulated with the excipient polyethoxylated castor oil, which induces hypersensitivity, nephrotoxicity and neurotoxicity (Singla et al., 2002). Nevertheless, the excellent antineoplastic properties of PTX are a redeeming factor, which has spurred interest in developing novel formulations for its delivery (Singla et al., 2002). In fact, many DDSs have been designed to deliver PTX to cancer cells (Liu et al., 2011). Recently, Liu et al. designed a DDS for PTX, which incorporates a molecular switch to enable cytosolic drug release through a switch-induced photothermal breakdown of the lysosome (Liu et al., 2021).

**FIGURE 6:**
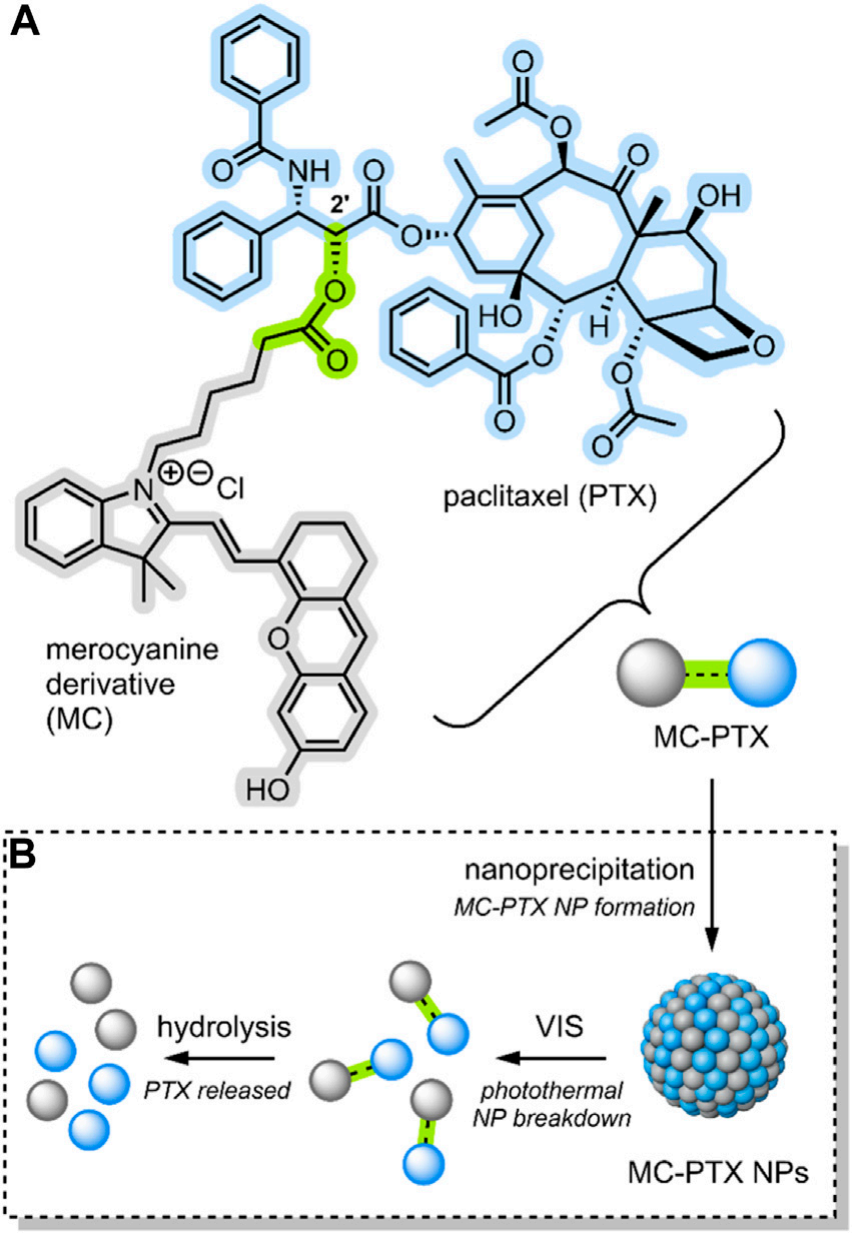
**(A)** An anthracene-like derivative of MC and PTX conjugated through an ester linkage at the 2′ position to form MC-PTX. **(B)** MC-PTX conjugates self-assemble into MC-PTX NPs through a nanoprecipitation process. VIS light irradiation of MC-PTX NPs results in NP breakdown and a rise in temperature (photothermal effect). The MC-PTX ester linkage is hydrolysed by cytosol enzymatic activity, resulting in PTX release (Liu et al., 2021). MC, PTX and the ester linkage have been highlighted grey, blue and green.

In 2021, Liu et al. reported the formation of a conjugate between PTX and an anthracene-type derivative of MC (MC-PTX) (Liu et al., 2021). In the MC-PTX conjugate, the MC was bound to the PTX through an ester linkage at the 2′ position (Figure 6A); this type of linkage is biodegradable by esterases overexpressed by cancer cells. Through nanoprecipitation, the group formed NPs comprised of their MC-PTX conjugate (Figure 6B).

Nanoprecipitation is a method designed to encapsulate hydrophobic drug molecules as spherical NPs, or inside nanocapsules (Martínez Rivas et al., 2017). Regarding the formation of spherical NPs, which is the nano-structure formed by Liu et al., the process requires two miscible solvents. The first solvent is one in which the hydrophobic drug is soluble; in this case, MC-PTX was solubilised in ethanol. The second, termed the non-solvent, is one in which the hydrophobic drug is not soluble; in this case, water. To form the NPs, the non-solvent is slowly combined with the solvent solution on stirring. As stirring continues, the solvent slowly evaporates, resulting in the precipitation of spherical NPs. To form nanocapsules, a film-forming material can be added to the solvent phase (Martínez Rivas et al., 2017). The overall nanoprecipiation process occurs as an effect of the complex interplay of flow, diffusion and surface tension between the solvent and the non-solvent (Bilati et al., 2005).

The MC-PTX NPs formed by Liu et al. had a spherical morphology; they were ∼110 nm in size, had a narrow distribution (<0.2 PDI), and were stable for 30 days in the dark. However, MC → SP isomerisation would occur when exposed to VIS light, destroying the nanostructures (Figure 6B). The isomerisation itself was evidenced by absorption spectroscopy. A temperature rise evidenced particle breakdown. In fact, studies involving the VIS light irradiation (638 nm) of MC-PTX NPs demonstrated a photothermal effect. The temperature increase rate was dependent on MC-PTX NP concentration and laser intensity.

As the MC molecular switch established VIS light control on the MC-PTX NPs, the team investigated the biological application of their system. Flow cytometry and confocal laser scanning microscopy were used to monitor the cellular uptake process of MC-PTX NPs into HeLa cells. Fluorescence intensified as NPs were incrementally added, indicating they are easily endocytosed. After the MC-PTX NPs were uptaken into the lysosomes, the cells were irradiated with VIS light (638 nm). As the MC-PTX NP nanostructures were destroyed due to MC → SP isomerisation, the temperature increased due to the photothermal effect. It was observed that this temperature increase resulted in the breakdown of the lysosomal membrane, thus releasing the MC-PTX conjugates into the cytosol. Finally, cell death followed. In this case, cell death is explained by esterase hydrolysis of the MC-PTX linkage, which results in the release of the potent antineoplastic PTX (Figure 6B).

Liu et al. successfully demonstrated a DDS incorporating a molecular switch that delivered a cytotoxic effect upon VIS light irradiation (Liu et al., 2021). Furthermore, the molecular switch enabled lysosomal escape by imparting photothermal control over the system. Thus, releasing the PTX and resulting in the cell’s inability to efflux the drug through its usual mechanism.

This section discussed the approach of incorporating a molecular switch into DDSs to impart light-responsive control over drug release. Specific case examples discussed involved the incorporation of azobenzene- and spiropyran-based switches to impart light-responsivity across the UV-VIS-NIR range of light. However, SPs are not limited in their application to light-stimulus only.

As discussed earlier, the MC isomer of SP is the energetically less favourable isomer, so it may not be stable in solution over time. Various methods can be employed to stabilise the MC form, such as the aforementioned use of a metal cation discussed in our studies (Baldrighi et al., 2016; Cardano et al., 2019). The MC isomer may also be stabilised by using a protic solvent, such as methanol, which will interact with the phenolate through hydrogen bonding to promote the open form. Furthermore, the pH value may be lowered to protonate the phenolate; hence, forcing an SP → protonated-MC (MCH, sometimes referred to as MCH^+^) conversion (Raymo and Giordani, 2001). The following section concerns the use of spiropyrans to impart dual, light and pH, control of drug release in DDSs.

## DDSs With Switches—Controlled by Light and pH

The first section of this review concerned cases where molecular switches were incorporated into DDSs to impart pH-induced control over drug release. The second section concerned cases where light was used as a stimulus for the same purpose. These two sections demonstrated how different components in a molecular switch could respond to a particular stimulus, which will trigger its switching mechanism. However, molecular switches are not necessarily limited to one single trigger. This section discusses cases where molecular switches, specifically spiropyrans, impart both light AND pH control over drug release in DDSs.

**FIGURE 7:**
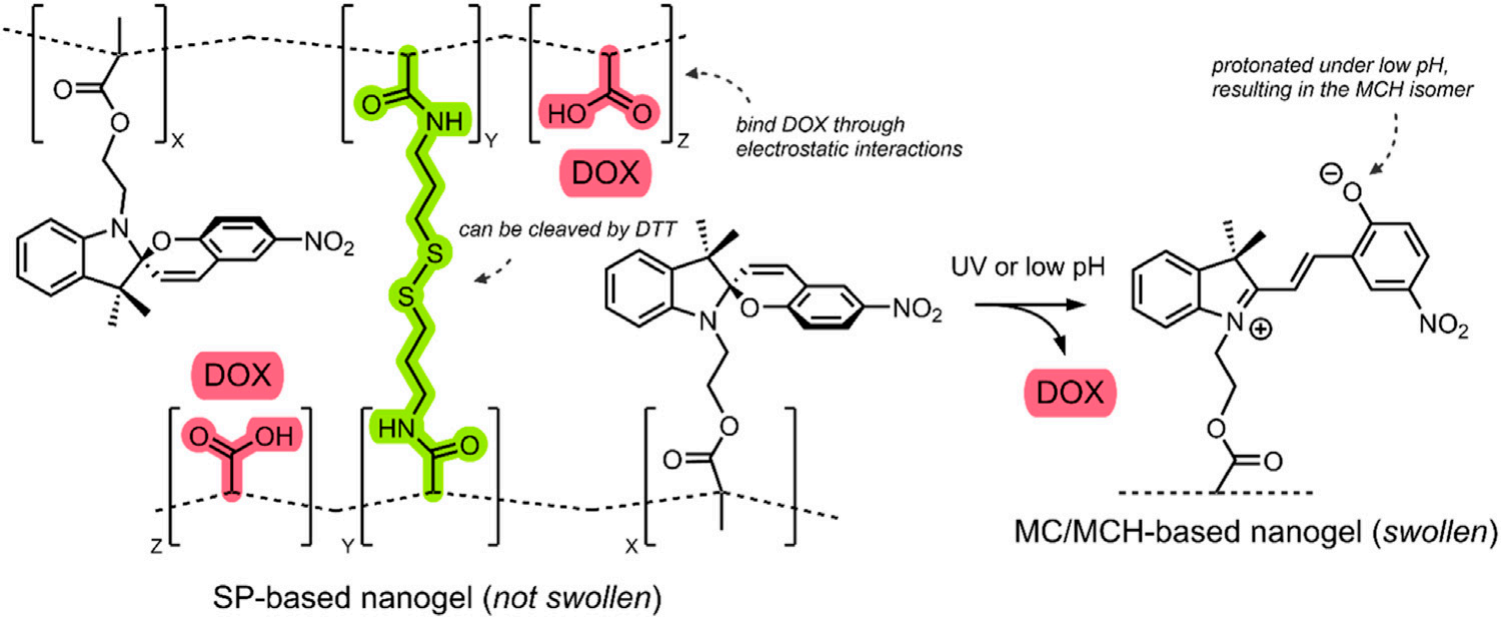
Schematic of the SP-based NG. The SP → MC/SP → MCH isomerisation is shown in response to UV/pH stimuli. The BAC crosslinker and the DOX-binding acrylic acids are highlighted green and red. The NG network was simplified with dashed lines (Chen et al., 2017).

In 2017, Chen et al. published an investigation of a triply-responsive nanogel (NG) that could be loaded with DOX through electrostatic interactions with acrylic acids (Chen et al., 2017). Through emulsion polymerisation, the group formed spherical NGs (∼40–60 nm) consisting of poly(acrylic acid-*co*-SP methacrylate) crosslinked by disulfide-containing *N*,*N*-bis(acryloyl)cystamine (BAC) (Figure 7) (Chen et al., 2017).

Incorporating an SP molecular switch into the system enabled UV light irradiation and low pH-induced control over disrupting the NG structure. Specifically, UV light irradiation (360 nm) induced the SP → MC isomerisation, and low pH resulted in SP → MCH isomerisation. Furthermore, incorporating a BAC crosslinker enabled the use of the dithiothreitol (DTT) reducing agent for the cleavage of the BAC crosslinkers, which was shown to occur through an oxidative scission (Chen et al., 2017). Transmission electron microscopy (TEM) studies of their NGs indicated that the UV, pH and redox-induced disruption of the NGs resulted in swelling. The small (40–60 nm) SP-based NGs swelled to 90–100 nm after UV irradiation (360 nm, 1 min, 15 mW/cm^2^), 250–350 nm under mildly acidic conditions (pH 6, 12 h), and 150–200 nm when exposed to a low concentration of DTT (4 mM, 12 h). This swelling indicates that the NGs could potentially release a therapeutic when exposed to the aforementioned stimuli (Chen et al., 2017).

To test the DTT-imparted redox and SP-imparted light and pH control over drug release, DOX was loaded into the system through water dissolution. UV-VIS absorption spectroscopy at 490 nm (λ_max_ of DOX) indicated that the DOX loading capacity of the NG was ∼18% w/w, which indicates that the DOX was bound to the acrylic acid groups in the NG network through electrostatic interactions. Chen et al. measured DOX release using a dialysis membrane and assayed it through UV-VIS absorption spectroscopy at different time intervals. Cumulative release profiles under the three stimuli individually and under a combination of the three were measured after a 24 h period (Chen et al., 2017).

After 24 h, under standard conditions (0 mM DTT, 0 min UV light irradiation, pH 7), a ∼11% DOX leakage from the DDS was observed. In the presence of the reducing agent, DOX release was found to be ∼41% at low (4 mM) DTT concentration and ∼66% at high (10 mM) DTT concentration; this release indicates that the SP-based NG DDS can release DOX in exposure to a reducing environment. When irradiated by UV light (360 nm, 1 min, 15 mW/cm^2^), a 52% DOX release was observed, which increased to 58% when irradiated for 3 min; this indicates that the SP imparts light-responsive control over drug release in their system. The most striking results were observed under decreasing pH. The ∼11% DOX release observed at pH 7 increased to ∼39% at pH 6 and ∼95% at pH 5. The group indicates that this pH-triggered DOX release is further aided by the protonation of SP molecular switch into the hydrophilic MCH form, and for the fact that DOX is bound to the acrylic acid groups by acid cleavable bonds (Figure 7). Chen et al. also investigated the synergistic effect of combined stimuli on drug release. The group exposed their SP-based NG DDS to pH 6, 1 min of UV irradiation, and 4 mM DTT, for 24 h. A DOX release of ∼80% was observed (Chen et al., 2017).

Overall, Chen et al. developed a triply-responsive SP-based NG DDS for DOX delivery responsive to UV light, low pH and redox stimuli. The group demonstrated the benefits of a combined effect of multiple stimuli. Efficient release was achieved without employing the harsh conditions of an extreme pH, long irradiation times or elevated concentrations of the reducing agent.

**FIGURE 8:**
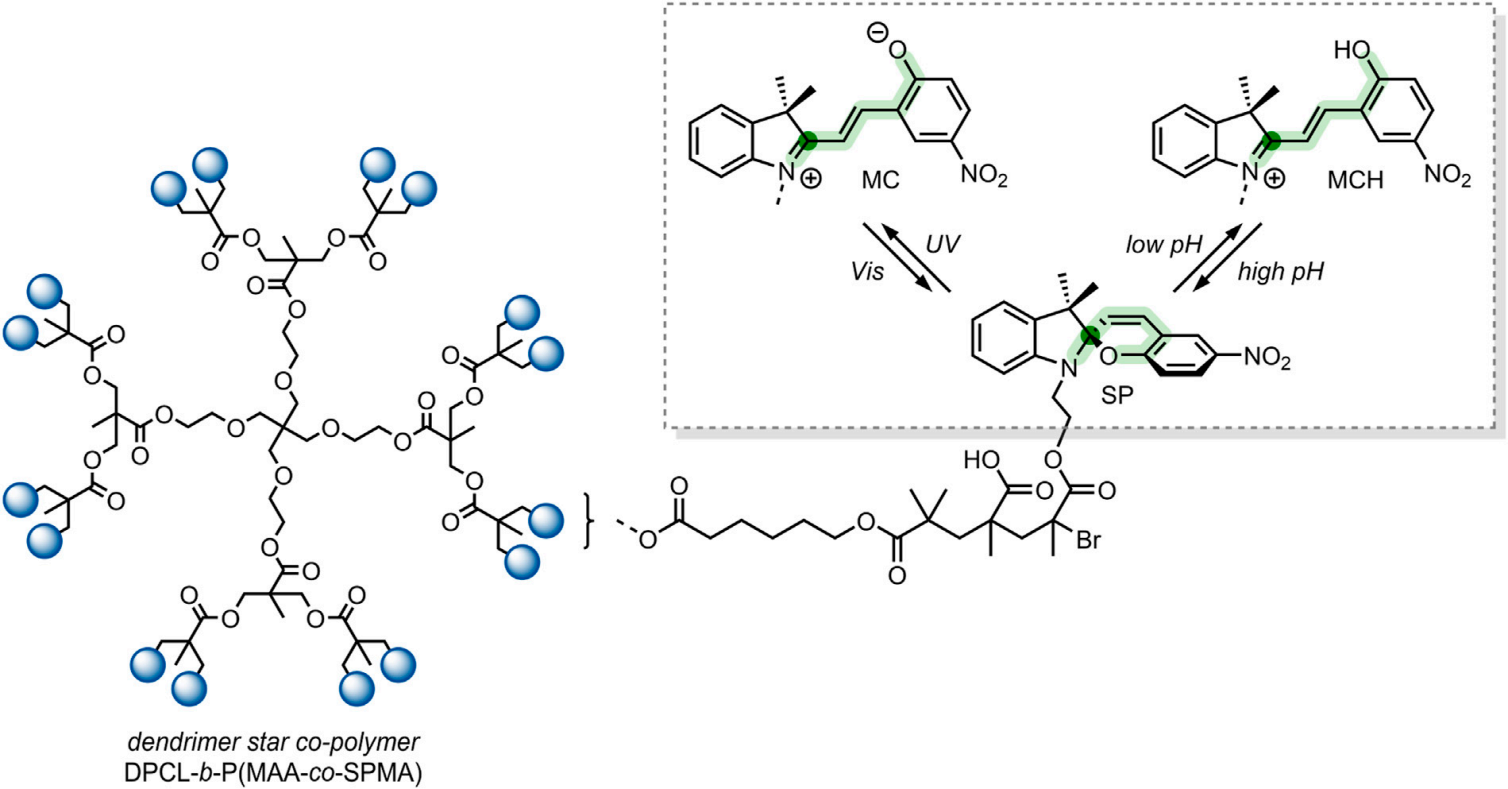
The structure of the DPCL-*b*-P(MAA-*co*-SPMA) complex, which self-assembles into micelles in an aqueous solution. *Framed*: UV light irradiation and low pH-induced isomerisation of the SP switch into the MC and MCH form, respectively (Yuan et al., 2018). The spiro-carbon is highlighted dark-green, and the SP chain is highlighted light-green.

In 2018, Yuan et al. reported on dually responsive dendrimer-star copolymers containing SP groups (Yuan et al., 2018). Dendrimers are highly branched, tree-like spherical compounds whose terminations can be designed using various chemical functionalities to aid in their solubilisation. The group produced an amphiphilic dendrimer-star copolymer poly(ε-caprolactone)-block-poly(methacrylic acid-co-spiropyran methacrylate) (DPCL-b-P(MAA-co-SPMA)) (Figure 8) through ring-opening polymerisation, atom transfer radical polymerisation and acidolysis (Yuan et al., 2018).

The group found that their dendrimer-star copolymer self-assembled into spherical micelles when exposed to an aqueous environment. The resulting micellar system was dually responsive to pH and UV light because of the SP molecular switch incorporated into the block co-polymer. SP underwent a ring-opening to form the MCH form when exposed to low pH, which was reversible under basic conditions (Figure 8). SP isomerisation also occurred in response to UV light, where SP isomerised into the MC form. The photochromic and acidochromic behaviour of the SP molecular switch used by Yuan et al. resulted in a morphological change of the micelle structure. When exposed to UV light or low pH, the P(MAA-*co*-SPMA) dendrimer branches underwent a conformational change, leading to a shift in the micelle morphology. Hence, the incorporation of a molecular switch established the possibility of utilising this micellar system for light and pH-controlled drug release (Yuan et al., 2018).

The group assessed the drug-delivery potential of their system using DOX as a model drug cargo. Through dialysis, DOX was encapsulated into the micelles, with a loading efficiency of ∼18% w/w. Then, the cumulative drug release was measured under varied conditions. DOX leakage after 18 h without irradiation, at pH 7.4, was ∼20%. The cumulative release after 18 h rose to 65% when the solution was irradiated with UV light (365 nm, 30 s). Moreover, when the pH was reduced to 6, the cumulative release approached ∼75% (Yuan et al., 2018). These results indicate that, while preferable to have varied stimuli to provoke drug release, certain systems may respond better to a particular stimulus over another. In this case, the irregular pH of the cancerous microenvironment may better induce drug release over exogenous UV light irradiation, which can have cytotoxic effects and low tissue penetration depending on the wavelength. Nevertheless, a DDS responsive to multiple stimuli is highly preferable as a dual application of both stimuli can have synergistic effects on drug release.

**FIGURE 9:**
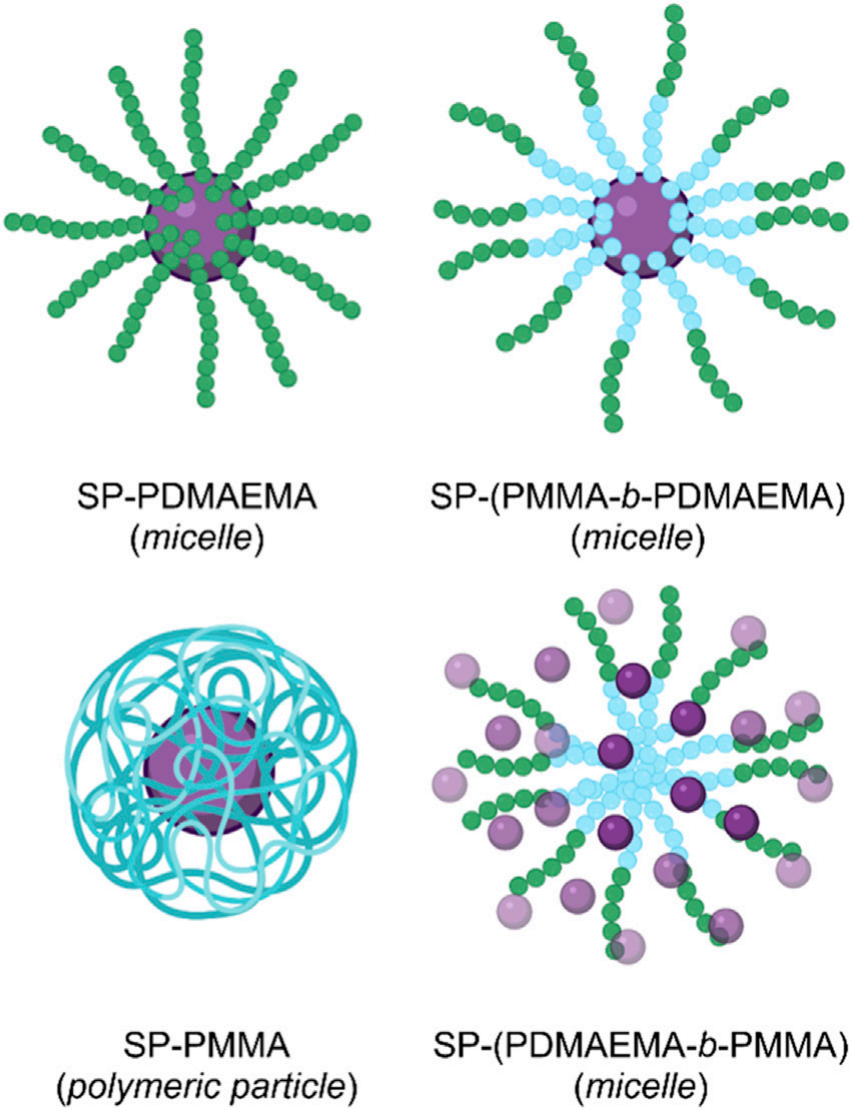
The four micellar and polymeric NPs assembled from the PMMA (blue) and PDMAEMA (green) block copolymers, with terminal SP (purple) groups (Razavi et al., 2020). Figure adapted with permission from Frontiers under a CC BY 4.0 license (Fagan et al., 2021).

The strategy of using SP-initiated block copolymer nanocarriers for DOX delivery was undertaken by Razavi et al. (2020). The group produced various block copolymers, which then self-assembled into micellar and polymeric nanostructures when exposed to aqueous solutions. The block copolymers constituted self-assembling hydrophilic poly(dimethylaminoethyl methacrylate) (PDMAEMA) with hydrophobic poly(methyl methacrylate) (PMMA) blocks, and the SP switch incorporated as a chain end group through atom transfer radical polymerisation. Four distinct polymers were formed; A) SP-PDMAEMA, B) SP-(PMMA-b-PDMAEMA), C) SP-(PDMAEMA-b-PMMA), and D) SP-PMMA,. Polymer A self-assembled into micelles with an SP core and a PDMAEMA corona. Polymer B self-assembled into micelles with a PDMAEMA corona and an SP-terminated PMMA core. Polymer C self-assembled micelles with an SP-terminated hydrophilic PDMAEMA corona and a hydrophobic PMMA core. Finally, polymer D self-assembled into polymeric NPs with an SP core and a PMMA outer shell (Figure 9) (Razavi et al., 2020).

To evaluate the effects of pH and light stimuli on their delivery systems, the group subjected each assembly to pH 5 and 9, with and without UV light irradiation (365 nm), for 5 min. As UV light and low pH stimuli induced SP isomerisation, the delivery systems were observed to swell. A similar swelling effect was observed by Chen et al. in their SP-based NGs, as discussed earlier (Chen et al., 2017). The swelling occurred due to water uptake by the system due to the formation of hydrophilic MC groups. Dynamic light scattering (DLS) was used to measure the resulting micellar swelling. Of the four SP-doped nano-structures produced by Razavi et al., the SP-PDMAEMA block copolymer micelles showed the most significant swelling in response to pH and light stimuli. At pH 5, these micelles increased from 340 to 530 nm, and the effect of UV light irradiation produced a change from 340 to 620 nm (Razavi et al., 2020).

Furthermore, the PDMAEMA polymer used by the group is thermally responsive, with a lower critical solution temperature (LCST) in the 30–60°C range. For this reason, the group investigated if the introduction of a molecular switch into the PDMAEMA polymer impacts its LCST. Remarkably, the group demonstrated that the SP molecular switch could control the thermal responsivity of the PDMAEMA polymer. The LCST of SP-PDMAEMA was measured to be 53°C. However, when irradiated with UV light, the resulting MC-PDMAEMA had an LCST of 60°C. This SP-imparted stimuli controlled swelling and thermal behaviour of the four nano-structures, highlights their potential use as controlled DDSs (Razavi et al., 2020).

Of their four polymer assemblies, the group investigated their three micellar-type polymer assemblies for their drug delivery potential. DOX was chosen as a model drug cargo and loaded onto the micelles through dialysis. The three micellar assemblies, A) SP-PDMAEMA, B) SP-(PMMA-b-PDMAEMA) and C) SP-(PDMAEMA-b-PMMA), had a final loading of ∼16%, ∼18% and ∼24% w/w, respectively. The cumulative release of DOX from these assemblies was subsequently measured over a 48 h period, under pH 5.3 and 7.4, at a temperature of 37 and 60°C, and with and without UV light irradiation (365 nm, 5 min, 6 W/cm^2^). The results of these DOX release studies are summarised in (Table 1). These results show the effects imparted by the SP molecular switch. All three assemblies show minimal release at pH 7.4 and 37°C. However, the micellar assemblies excellent DOX release when exposed to pH 5.3 or UV light irradiation. Furthermore, the thermal effects of the PDMAEMA polymer are also evident, as the micellar system undergoes dissolution at 60°C (the LCST of the polymer), resulting in drug release (Razavi et al., 2020).

**TABLE 1:** DOX release measured after 48 h from the three micellar polymer assemblies; A) SP-PDMAEMA, B) SP-(PMMA-*b*-PDMAEMA) and C) SP-(PDMAEMA-*b*-PMMA) (Razavi et al., 2020).

	A (%)	B (%)	C (%)
pH 7.4, 37°C	20	33	15
pH 5.3, 37°C	75	89	73
pH 7.4, 60°C	86	94	84
pH 7.4, 37°C, UV	97	98	94

Overall, Razavi et al. demonstrated the formation of four different polymer assemblies with two types of SP-doped polymers. All four assemblies were responsive to pH and light stimuli, as imparted by the SP molecular switch. Three micellar polymer assemblies were tested for their drug delivery application, and all showed excellent stimuli induced release. Interestingly, it was observed that the SP molecular switch could control the LCST of a thermal polymer. This control was enabled by the hydrophilicity contrast between the two isomeric forms of the molecular switch (Razavi et al., 2020).

Different DDSs with dual pH and light-responsive drug release control were discussed in this section. There are clear advantages of having dual stimulus control over drug release. Depending on the type of cancer, particular tumour microenvironments may exhibit a natural pH, in which case light can be used to control drug release. Similarly, in cases where light cannot penetrate the tissue, a DDS switch may rely on the specific pH of the tumour microenvironment to induce conformational change and subsequent drug release. Furthermore, combined stimuli may synergistically induce a higher % drug release than a single stimulus can. The polymer-assembly DDSs designed by Razavi et al. were found to have a switch-induced control over a polymers LCST, and consequently, the temperature at which drug release may occur. Whilst thermal responsivity may not necessarily be as viable as light and pH as a trigger for controlled drug release, it is interesting to see the other types of control a molecular switch may impart on a DDS.

## Conclusion and Perspectives

The investigation and application of the unique properties of molecular switches have been an area of interest from midway through the last century. Still, recent developments in material science and nanotechnology have enabled their use in the biomedical field. Topically, molecular switches are commonly incorporated into nanocarriers, nano-sized vesicles designed to deliver drugs in a targeted manner. These nanocarriers offer a solution to the increasing issue of poor solubility and membrane permeability of conventional chemotherapeutics, the MDR effect, and systemic toxicity. These nanocarrier systems typically comprise a nano-structure encapsulating a therapeutic for targeted and controlled release.

The influence of these nanocarriers’ bio-compatible controlled drug-release mechanism cannot be overstated in improving the selectivity and efficacy of APIs. The ability of a nanocarrier to deliver a therapeutic to a target site and a mechanism to induce tightly controlled release can minimise undesired side effects and enhance drug potency.

This review outlined how this controlled drug-release mechanism can be imparted on DDSs through the incorporation of molecular switches. Recent developments in DDS systems incorporating molecular switches for controlled drug release were presented and discussed. A focus was placed on systems where exogenous control using light as a stimulus and endogenous control using pH changes as a stimulus were enabled. These stimuli were discussed for their particular accessibility, spatiotemporal control, and viability regarding anticancer drug delivery. A summary of the different DDSs discussed herein has been presented in (Table 2). The emerging interest area of using molecular switches to impart light and pH-responsive drug-release control on DDSs is promising. However, these systems face particular challenges.

**TABLE 2:** Summary of herein reviewed delivery systems incorporating molecular switches for light- and/or pH-responsive payload release. Molecular switches have been visualised as they would appear prior to activation and prior to formation of larger nanostructures. Delivery payload has been visualised is as it would appear after release, cleavage, or activation. The sequence for the siRNA example has been sourced from (Eckstein, 2005).

System control imparted by switch	Molecular switch	Delivery payload	References
**System**	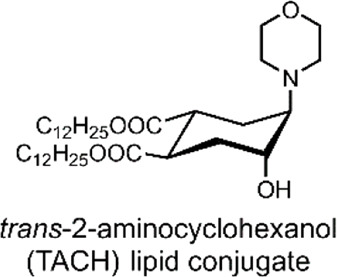	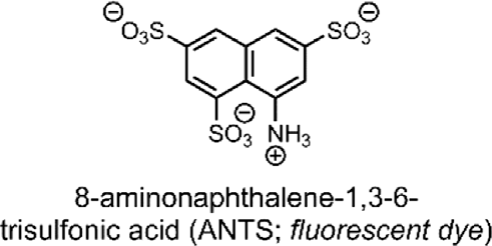 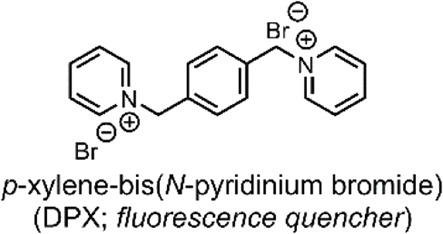	Brazdova et al. (2008)
Liposomal delivery system incorporating a lamellar switch for pH-responsive payload release **pH-Triggered Payload release**			
The ANTS/DPX liposomal content is released through mildly acidic pH-driven chair conformation ring flip of the molecular TACH-lipid switch, and subsequent liposomal destabilisation due to the lipid tails shifting from an equatorial to axial position			
*For more information, see: (* *Figure 1* *)*			
**System**	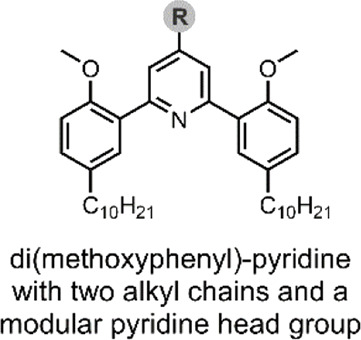	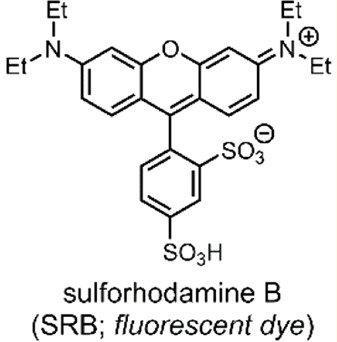 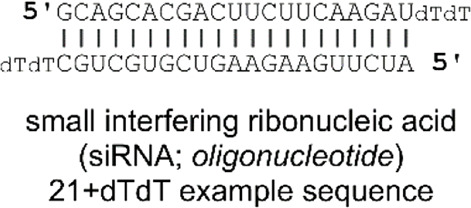	(Viricel et al., 2015, 2017)
Liposomal DDSs (<∼200 nm) incorporating a lamellar switch for pH-responsive payload release into the cytosol			
**pH-Triggered Payload release**			
Liposomal content released through low-pH driven lipid-tail rotation around the alkylated di(methoxyphenyl)-pyridine molecular switch (C_pyr_—C_phe_ bond rotates), thus inducing liposomal structure reorganisation and subsequent liposome dissolution, releasing the SRB or siRNA payload			
*For more information, see: (* *Figure 2* *)*			
**System**	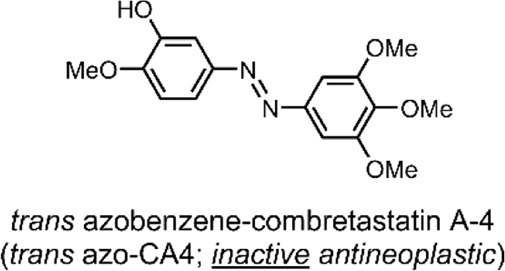	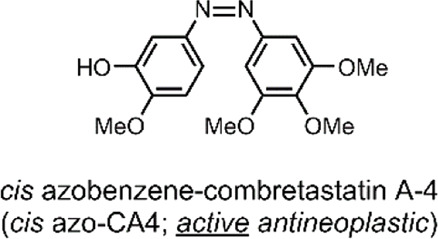	Sheldon et al. (2016)
Difunctional molecule with UV-responsive therapeutic activation			
**UV-Triggered Therapeutic Activation**			
The pharmaceutically inactive *trans* azo-CA4 isomerises to the active *cis* form on UV light irradiation			
*For more information, see: (* *Figure 3* *)*			
**System**	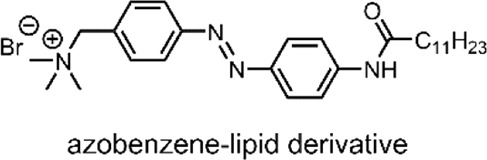	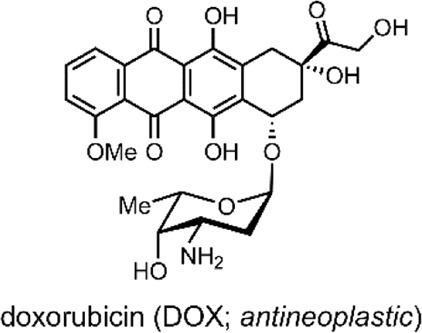	Yao et al. (2016)
Liposomal DDS (∼200 nm) doped with UCNPs and incorporating a lamellar switch for NIR-responsive drug release			
**NIR-Triggered Payload release**			
On NIR light irradiation, UCNPs generate UV/VIS light, resulting in repeated azonbenzene isomerisation, thus inducing lipiposomal destabilisation and DOX release			
*For more information, see: (* *Figure 4* *)*			
**System**	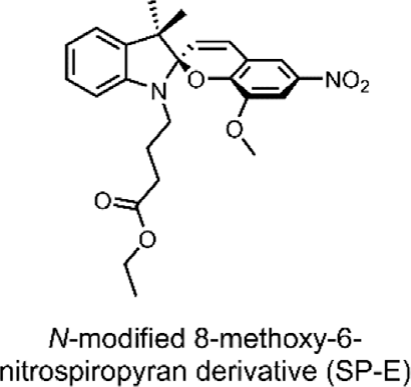	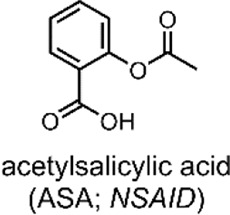	Cardano et al. (2019)
Supramolecular ternary complex DDS with switch chelate for VIS-responsive drug release			
**VIS-Triggered Payload release**			
VIS light irradiation of the MC-Zn^2+^-ASA ternary complex results in MC → SP isomerisation and ASA release (as the SP is unable to chelate with Zn^2+^ to stabilise the complex)			
*For more information, see:* (*Figure 5* *)*			
**System**	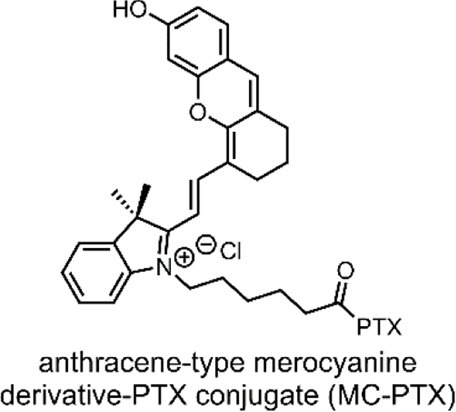	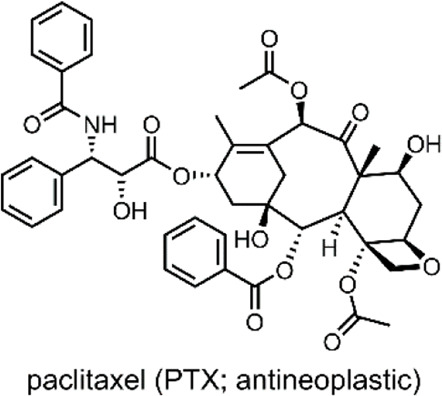	Liu et al. (2021)
NP DDS (∼110 nm, spherical) comprised of switch-drug conjugates for VIS-responsive lysosomal escape and payload release			
**VIS-Triggered Payload release**			
VIS light irradiation of the MC-PTX NPs after endocytosis results in MC → SP isomerisation, which results in NP breakdown and a temperature rise (photothermal effect), which breaks down the lysosomes, hence releasing the and MC-PTX payload. Esterases break the MC-PTX linkage, releasing PTX			
*For more information, see: (* *Figure 6* *)*			
**System**	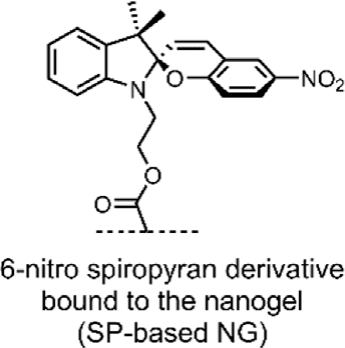	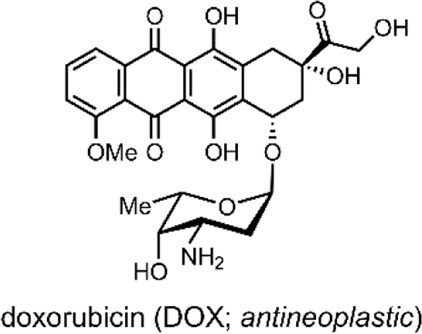	Chen et al. (2017)
NG DDS incorporating a switch for UV or pH responsive drug release, and disulfide linkers for redox sensitive drug release			
**UV or pH-Triggered Payload release**			
UV light irradiation or low pH results in the swelling of the NG due to SP → MC isomerisation, resulting in DOX release (*Note: the NG also incorporates disulfide linkers, which can be cleaved by DTT, resulting in DOX release*)			
*For more information, see: (* *Figure 7* *)*			
**System**	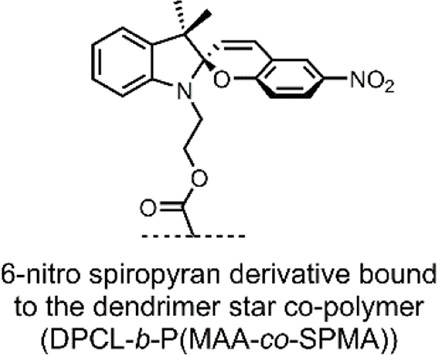	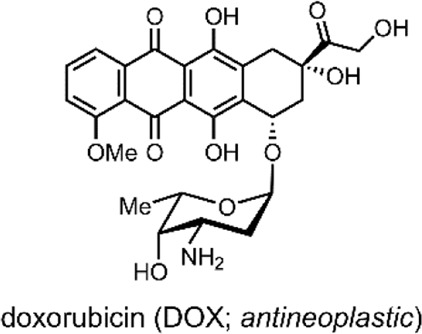	Yuan et al. (2018)
Micellar DDS incorporating a switch for UV or pH-responsive drug release			
**UV or pH-Triggered Payload release**			
UV light irradiation or low pH results in SP → MC isomerisation, inducing comformational changes in the dendrimer branches, altering micellar morphology, resulting in DOX release			
*For more information, see: (* *Figure 8* *)*			
**System**	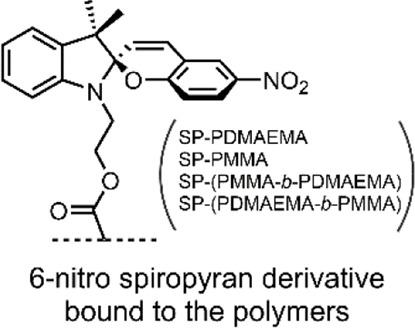	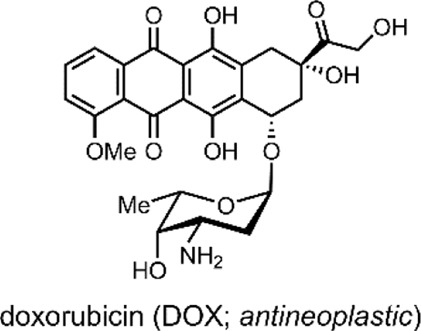	Razavi et al. (2020)
Micellar and polymeric NP DDSs with pH- and UV-responsive payload release			
**UV or pH-Triggered Payload release**			
UV light irradiation and low pH induce micellar swelling due to SP → MC isomerisation, which results in DOX release			
*For more information, see: (* *Figure 9* *)*			

For instance, UV light irradiation is required to induce conformational changes in molecular switches such as SPs and azobenzenes. This ionising energy source has wavelength-dependent cytotoxicity and minimal tissue penetration depth. NIR light is a viable alternative as it is biologically safe with good tissue penetration. However, NIR light does not have sufficient energy to trigger a molecular switch. Nevertheless, innovative solutions are being developed to overcome this issue. In this review, we have seen how the incorporation of UCNPs within a DDS can enable a switch-induced drug release through NIR-light stimulation.

Another issue involves the cellular degradation of the DDS before a therapeutic release at the active site. DDSs entering the cell through an endosomal pathway may end up in the lysosome, where they will be degraded through enzymatic activity. This review shows how molecular switches incorporated into NPs can elicit a photothermal effect, resulting in lysosomal breakdown and subsequent drug release into the target site.

In some instances, the application of a stimulus to a switch-modified DDS may not result in sufficient drug release. This review presented a number of systems with dual stimuli-responsive molecular switches. Dual stimuli control over drug release is of particular benefit as the synergistic effect may result in increased drug release under milder stimuli conditions.

Overall, molecular switches have been shown as viable components for imparting drug-release control in DDSs. The interesting solutions molecular switches offer to the above-mentioned challenges inspire their further study and use in DDSs.

## Glosssary

λmax: absorption maxima wavelength
ANTS: 8-aminonaphthalene-1,3,6-trisulfonic acid
API: active pharmaceutical ingredient
ASA: acetylsalicylic acid
azo-CA4: azobenzene analogue of CA4; BAC=N,N-bis(acryloyl)cystamine
CA4: combretastatin A-4 phosphate
CD44: cluster of differentiation 44
COX: cyclooxygenase
DC: direct-current
DDS: drug delivery system
DLS: dynamic light scattering
DTT: dithiothreitol: dithiothreitol; EC50=half maximal effective concentration
DNA: deoxyribonucleic acid
DMSO: dimethyl sulfoxide
DOTAP: dioleoyl-3-trimethylammonium propane
DOX: doxorubicin
DPCL-b-P(MAA-co-SPMA): dendrimer-star copolymer poly(ε-caprolactone)-block-poly(methacrylic acid-co-spiropyran methacrylate)
DPX: p-xylenebis-pyridinium bromide
DSPC: 1,2-distearoyl-sn-glycero-3-phosphocholine
DSPE-PEG2000: 1,2-distearoyl-sn-glycero-3-phosphorylethanolamine-N-[amino(polyethylene glycol)-2000]
DTT: dithiothreitol: dithiothreitol; EC50=half maximal effective concentration
EPR: enhanced permeability and retention
GSH: glutathione
HeLa: Henrietta Lacks cervical cancer immortal cell line
HUVEC: human umbilical vein endothelial cell line
LC-MC: liquid chromatography–mass spectrometry
LCST: lower critical solution temperature
LED: light-emitting diode
MC: merocyanine
MCH: protonated MC
MDR: multidrug resistance
NG: nanogel
NIR: near infrared
NMR: nuclear magnetic resonance
NOESY: nuclear Overhauser effect spectroscopy
NP: nanoparticle
PDMAEMA: poly(dimethylaminoethyl methacrylate)
PMMA: poly(methyl methacrylate)
POPC: 1-palmitoyl-2-oleoyl-syn-glycero-3-phosphocholine
PTX: paclitaxel
ROS: reactive oxygen species
siRNA: small interfering ribonucleic acid
SP: spiropyran
SP-E: N-modified 8-methoxy-6-nitrospiropyran
SRB: sulforhodamine B
SWIR: short wave infrared
t½: half-life
TAB: trimethyl ammonium bromide
TACH: trans-2-aminocyclohexanol
TEM: transmission electron microscopy
UCNP: upconversion nanoparticles
UV: ultraviolet
UVA: ultraviolet A
VIS: visible.
